# A Unified Microscopic Picture of Cation and Anion Migration in MAPbI_3_

**DOI:** 10.1021/acs.jpclett.6c02367

**Published:** 2026-10-15

**Authors:** Viren Tyagi, Geert Brocks, Shuxia Tao

**Affiliations:** † Department of Applied Physics and Science Education, 3169 Eindhoven University of Technology, 5600 MB Eindhoven, The Netherlands; ‡ Computational Chemical Physics, Faculty of Science and Technology and MESA+ Institute for Nanotechnology, 3230 University of Twente, 7500 AE Enschede, The Netherlands

## Abstract

Passivating defects and restricting defect mobilities in halide perovskites to increase device lifetimes has become a main field of research. Modeling structure and mobility of point defects is an essential contribution to this endeavor. We employ molecular dynamics, based on neural network potentials trained on density functional theory data, to model ion migration in MAPbI_3_ triggered by I and MA vacancies or interstitials. Most of these species diffuse rapidly at room temperature, with migration barriers between 0.15 and 0.20 eV. MA interstitials are highly mobile despite their molecular nature, because of a collective reorientation of neighboring MA ions. No evidence of MA vacancy migration is obtained. While the diffusion of I-related defects appreciably depends on their charge state, the diffusion of MA defects does not. These results revise the conventional picture of ion transport in hybrid perovskites and highlight the role of collective molecular motion in enabling fast ionic migration.

## Graphical abstract

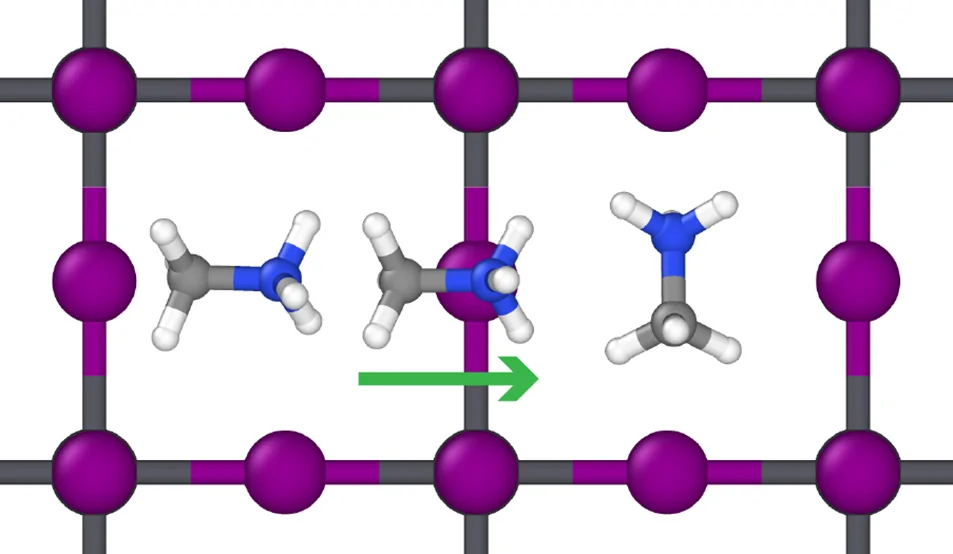

Hybrid organic–inorganic metal halide perovskites have shown great potential for application in photovoltaic and light-emitting devices.^1^ The materials allow for optimization of optoelectronic properties through chemical compositional flexibility and low-temperature solution processing.^2,3^ Halide perovskites are soft materials, however, where defects in the crystal lattice are relatively easily formed.^4,5^ Whereas most of these defects do not seem to alter the basic optical and electronic parameters much, they can trigger chemical decomposition and degradation.^6^ Defects facilitate ion migration through the material to surfaces, grain boundaries, and interfaces with electrodes, where degradation reactions are likely to take place.^7,8^ Passivating defects or limiting defect mobilities in order to enlarge device lifetimes has become a main field of research in halide perovskites.^9,10^ Identifying the character and mobility of the relevant migrating defects is then the first essential step.

A majority of experimental studies on ion transport in halide perovskites focus on MAPbI_3_ (MA = CH_3_NH_3_^+^, methylammonium) as the prototype halide perovskite material. Assuming that the observed ion migration behaviors relate to intrinsic properties of bulk MAPbI_3_, the prime vehicles for diffusion should be point defects, i.e., single ion interstitials and/or vacancies, rather than extended or compound defects.^11^ It is generally agreed upon that Pb interstitials and vacancies are immobile at room temperature, which leaves I and/or MA defects as possibilities.^12,13^

Halide anion migration receives most attention in MAPbI_3_. Temperature-dependent measurements allow for the extraction of migration barriers, but the numbers extracted from different experiments show a considerable spread. Experiments cover (temperature-dependent) conductivity, capacitance or photoluminescence,^14–25^ transient ion drift,^26^ impedance and deep-level transient spectroscopy,^27–31^ and find diffusion barriers ranging from 0.10 to 0.68 eV. Some of these experiments also report barriers for the diffusion of MA cations, with again a considerable spread in numbers from 0.18 to 0.94 eV. Per study the numbers for the migration barriers of MA cations tend to be somewhat larger than those for the I anions, but both species can be mobile.

Whereas charge states, energy levels, and diffusion coefficients of migrating species are experimentally accessible, the nature and composition of the actual migrating defects are less so. Studies therefore use density functional theory (DFT) calculations as support. The workhorse method for extracting migration barriers is based on transition state theory (TST) and finding a lowest-energy path following the nudged elastic band (NEB) method, for instance. This approach suffers from the difficulty of finding such a path in a soft material such as MAPbI_3_, where in fact many such paths may compete and contribute, as the low symmetry of the MA cation enables multiple energetically competitive configurations.^32,33^ Values reported for the migration barriers range from 0.08 to 0.58 eV for the iodide vacancy, 0.08 eV for the iodide interstitial, 0.38 to 0.48 for the MA interstitial, and 0.46 to 0.96 eV for the MA vacancy.^15,34–39^

Migration rates or diffusion coefficients cannot be extracted directly from a NEB calculation. This is possible using molecular dynamics (MD) simulations. Molecular dynamics samples all relevant migration pathways and configurations. It allows for direct simulations of ion diffusion at preset temperatures, giving access to diffusion rates and barriers. The Achilles heel of MD is the force field used in the simulations. From a parametrized analytical force field, migration barriers of 0.1 and 0.55 eV have been extracted for the iodide vacancy and interstitial, respectively.^40,41^ The accuracy of such force fields is debated, however.^33^ A breakthrough has been achieved in recent years in the form of machine learned force fields (MLFFs), which allow for MD simulations using force fields at DFT accuracy.^42–44^ By selecting the DFT data on which the MLFFs are trained, it is possible to target MD simulations toward specific charge states of the migrating species.^45,46^

We use MD based on MLFFs to model ion migration in MAPbI_3_ triggered by I anion vacancies or interstitials, and by MA cation vacancies or interstitials. We find all species to be extremely mobile, except for the MA vacancy. The mobility of halide anion defects, vacancies and interstitials, is comparable to that found in the all inorganic compounds CsPb­(I_*x*_Br_1–*x*_)_3_; *x* = 0, ..., 1,^45,47^ leading to the conclusion that the A cation species in the perovskite structure does not affect halide motion much. Remarkably, the diffusion rate of MA interstitial cations is only slightly smaller than that of I interstitial anions. Migration of MA interstitials proceeds via a collective reorientation of the interstitial and its neighboring MA ion in the lattice. Whereas the diffusion of I-related defects very much depends on their charge state, that of MA-related defects is virtually independent of charge state. We attribute this to the fact that an MA cation interstitial or vacancy only create very shallow impurity levels that are ionized at room temperature.

Iodide and MA interstitials and vacancies have charge states I_I_^–^, V_I_^+^, I_MA_^+^, and V_MA_^–^, respectively, under intrinsic or mildly doped conditions.^48,49^ Results obtained from DFT calculations differ somewhat on whether vacancies or interstitials are predicted to be the dominant species under equilibrium conditions. This reflects the use of different functionals, for instance.^48,50^ The formation energy of interstitial-vacancy (Frenkel) pairs I_I_^–^/V_I_^+^ and I_MA_^+^/V_MA_^–^ only weakly depends on the functional,^50^ however, including Van der Waals (VdW) interactions favors interstitials over vacancies, whereas excluding VdW interactions favors vacancies, demonstrating that VdW interactions have to be included.^48,50^

In this paper, we consider both vacancies and interstitials and train a MLFF for describing them in their most prominent charge states under intrinsic conditions.We have chosen for a MLFF in this case over ReaxFF^51^ for instance, as it allows for achieving accuracy more straightforwardly. Our MLFF is based on the Allegro model, which is a local E(3)-equivariant graph neural network potential (NNP), as implemented in the NequIP package.^52,53^ The NNP has the advantage over a SOAP/GAP MLFF that regarding computational costs it scales much better with the number of atomic species.^45,47^ The Allegro model uses a cutoff radius, which may seem to exclude a proper description of long-range electrostatic interactions. It has been shown in numerous studies, however, that to capture ionic motion and ion migration in the type of material we are considering here, short-range interactions suffice.^53–55^

The data sets for training and validating the network are generated using the on-the-fly module as implemented in VASP, sampling structures from short-time scale MD runs.^56–60^ Energies, forces, and stress tensors are calculated using DFT with the SCAN+rVV10 functional.^61,62^ These are calculated for a series of structures that are sufficiently different from one another, using selection criteria based on Bayesian inference. The training and validation sets are generated using 2 × 2 × 2 cubic supercells MAPbI_3_ (96 atoms) containing one charged or neutral MA or I point defect. Details of the training procedure are explained in the Supporting Information (SI) (note 1).

Iodide vacancies (interstitials) give donor (acceptor) levels that can be populated with electrons (holes) if the (quasi) Fermi level is very close to the conduction (valence) band, which might happen under device operating conditions.^63,64^ The same is true in principle for MA interstitials and vacancies. A NNP trained on energies and forces has no direct knowledge of electronic structure, but it is possible to select DFT data for training a neural network that are generated with a Fermi level in or close to the valence or conduction band to represent defects with modified charge states. In this way, we train separate NNPs for I_MA_^0^ and V_MA_^0^.

A standard test for MLFFs is to compare migration barriers along paths generated using the climbing image nudged elastic band (CI-NEB) technique with those calculated by DFT along the same paths.^65^ The problems NEB has in establishing migration barriers in halide perovskites were mentioned in the introduction, but for the sake of testing, there is no need to accurately determine the lowest energy path(s); any reasonable path showing a migration event will do. The computed energies along NEB paths are given in Figure 1, and the computed NEB barriers in Table 1. The difference between the NEB barriers calculated using DFT and the NNP (|Δ*E*_b_^DFT^ – Δ*E*_b_^NNP^|) is less than 0.11 eV in all cases, indicating the accuracy of the NNPs. No migration events were actually observed during training for the neutral MA vacancy, V_MA_^0^, yet the NNP accurately describes the NEB barrier for this defect (|Δ*E*_b_^DFT^ – Δ*E*_b_^NNP^| = 0.02 eV) (Figure 1c), demonstrating the accuracy of the NNP in extrapolating to events not explicitly included in training.

**Figure 1:**
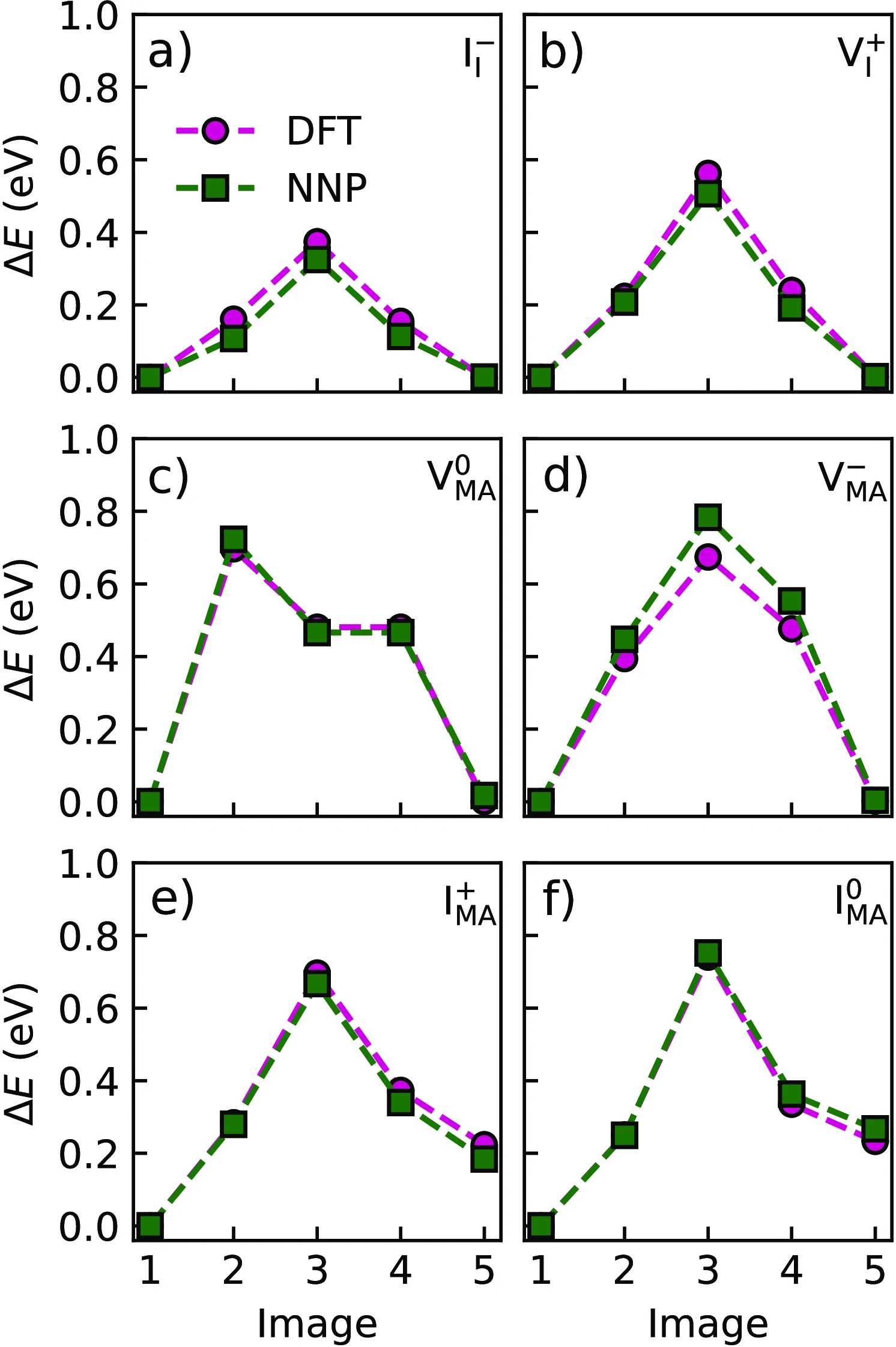
Energies along the NEB migration paths, calculated using DFT and the NNP for (a) negatively charged iodide interstitial (I_I_^–^), (b) positively charged iodide vacancy (V_I_^+^), (c) neutral MA vacancy (V_MA_^0^), (d) negatively charged MA vacancy (V_MA_^–^), (e) positively charged MA interstitial (I_MA_^+^) and (f) neutral MA interstitial (I_MA_^0^). The points are the calculated values, and the lines guide the eye; the energy of the minima is set to 0.

**Table 1:** NEB Barriers (*E*_b_) Calculated Using DFT and the NNPs for the NEB Migration Paths

System	*E*_b_^DFT^ (eV)	*E*_b_^NNP^ (eV)
I_I_^–^	0.37	0.33
V_I_^+^	0.56	0.51
V_MA_^0^	0.70	0.72
V_MA_^–^	0.67	0.78
I_MA_^+^	0.70	0.67
I_MA_^0^	0.74	0.75

For I_MA_^+^ (Figure 1e) and I_MA_^0^ (Figure 1f), the initial and the final images do not have the same energy, indicating that these are not the complete migration paths. It illustrates the problem of finding the lowest energy migration path in this compound. As MA cations are asymmetric and polar, the local structure around a migrating I defect allows for multiple local minima, and significant hysteresis. In fact, the NEB barriers appreciably overestimate the migration barriers as they are extracted from the MD simulations, as we will see below. The details of the CI-NEB calculations along with the geometries of the starting, saddle and final points along the CI-NEB paths for all defects are given in the SI (note 2).

The NNP is trained on total energies, forces, and stress tensors. One can test these quantities produced with the NNP against values calculated using DFT. This is done using structures sampled from MD simulations at 600 K performed on 6 × 6 × 6 supercells of MAPbI_3_ (2592 atoms) with one I or MA point defect. From these comparisons, for total energies the coefficient of determination *R* ^2^ ≥ 0.84 and the mean absolute error (MAE) ≤ 0.32 meV/atom, for the forces *R* ^2^ = 0.99 and MAE ≤ 49.06 meV/Å and for the elements of the stress tensor *R* ^2^ ≥ 0.96 and MAE ≤ 0.12 kB. Details about this test can be found in SI (note 2). Finally, the stability of the force fields is validated by computing the energy drift in NVE simulations, the details of which can also be found in SI (note 2).

Following training and validation, the NNPs are used to perform three independent 2 ns long MD simulations at each temperature for five temperatures between 500 and 600 K. The details of these production runs are given in the SI (note 4). From these simulations, diffusion coefficients are extracted, providing the temperature-dependent diffusion behavior. As ion transport in MAPb_3_ via interstitials or vacancies takes place through kick-out processes, tracer diffusion is not appropriate for characterizing transport. Instead, we include all atoms or molecules of a particular species in calculating the diffusion coefficient, using the Einstein relation, which links the slope of the mean square displacement to time:

1

$$D=\frac{1}{6}\;\underset{t\to \infty }{\lim}\frac{d}{dt}{\left\langle \overset{N}{\underset{i=1}{\sum }}{∥r_{i}\left(t+t_{0}\right)-r_{i}\left(t_{0}\right)∥}^{2}\right\rangle }_{t_{0}};,t>t_{0}$$

where *N* is the number of atoms of a particular species in the simulation box, and **r**_*i*_ are the atomic positions. Notwithstanding the fact that all ions participate in extracting the diffusion coefficient *D*, in the following, we will still label them according to the defect, interstitial, or vacancy that instigates the diffusion. All mean squared displacement plots and diffusion coefficients are available in ref 66.

The diffusion coefficients *D* extracted from the simulations are given in Figure 2. For iodide interstitial I_I_^–^ and vacancy V_I_^+^, the diffusion coefficients are within a factor of 5 of each other within the given temperature range, with the interstitial being the more mobile of the two. Remarkably, also the diffusion coefficients of the neutral and positive MA interstitials I_MA_^0^, I_MA_^+^ are within the same range. No migration events are observed for the MA vacancies V_MA_^0^, V_MA_^–^ in the simulated temperature and time ranges.

**Figure 2:**
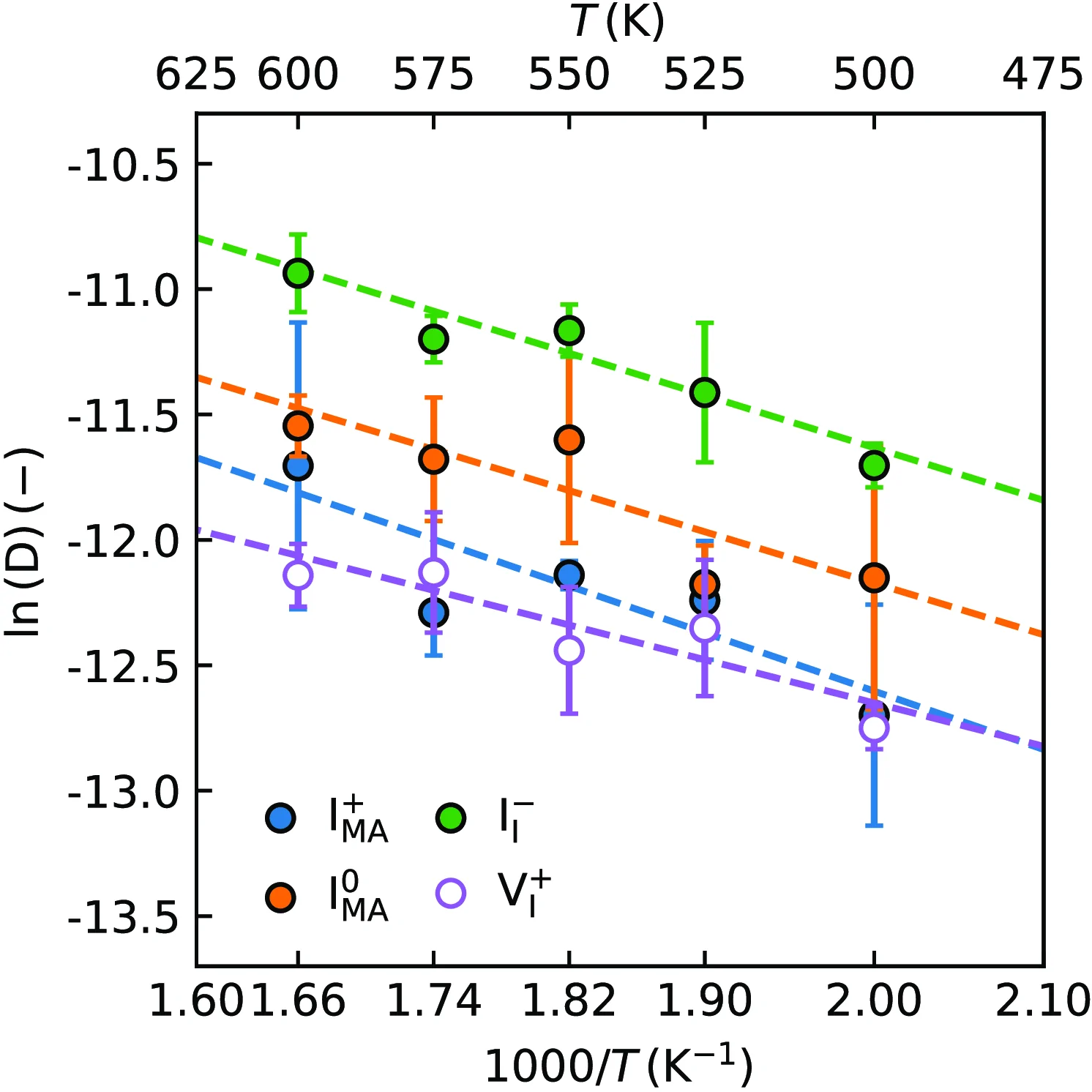
Temperature-dependent diffusion coefficients of iodide interstitial (I_I_^–^), iodide vacancy (V_I_^+^), neutral MA interstitial (I_MA_^0^), and positive MA interstitial (I_MA_^+^) defects in MAPbI_3_. The dashed lines represent the fits to an Arrhenius expression, and the error bars represent the standard error in the mean at each point.

For all defects, the temperature dependence of the diffusion coefficient can be fitted to an Arrhenius relation,

2 $$D=D_{0}\;\exp\left(-\frac{E_{a}}{k_{B}T}\right)$$

where *k*_B_ is the Boltzmann constant, *E*_a_ the activation energy, and *D*_0_ the pre-exponential factor. The fits are given in Figure 2, and the fit parameters along with the extrapolated diffusion coefficient at room temperature (*D*_300 K_) are presented in Table 2.

**Table 2:** Activation Energies (*E*_a_) and Pre-exponential Factors (*D*_0_) Extracted from the Arrhenius Fits, And Extrapolated Diffusion Constants (*D*_300 K_) at Room Temperature for Iodide Interstitial (I_I_^–^), Iodide Vacancy (V_I_^+^), Neutral MA Interstitial (I_MA_^0^), and Positive MA Interstitial (I_MA_^+^) in MAPbI_3_

System	*E*_a_ (eV)	*D*_0_ (× 10^–4^ cm^2^ s^–1^)	*D*_300 K_ (× 10^–7^ cm^2^ s^–1^)
I_I_^–^	0.18 ± 0.03	5.93 ± 3.97	5.61
V_I_^+^	0.15 ± 0.04	1.04 ± 0.92	3.19
I_MA_^0^	0.18 ± 0.06	3.16 ± 4.27	2.99
I_MA_^+^	0.20 ± 0.07	3.47 ± 5.12	1.51

The activation energies and prefactors for interstitial I_I_^–^ and vacancy, V_I_^+^ are quite close, and their extrapolated diffusion coefficients at 300 K are within a factor of 2. The numbers are also quite close to those found for halide interstitials in CsPbI_3_, CsPbBr_3_ and CsPb­(I_*x*_Br_1–*x*_)_3_,^45,47^ indicating that halide diffusion in 3D perovskites does not depend too much on the A cation in the lattice.

Diffusion coefficients for MA interstitials have similar prefactors as those for I defects, and only slightly higher activation energies, which demonstrates that MA interstitials are as mobile as I defects. In addition, the charge state of the MA interstitial does not seem to impact its migration behavior much. A neutral MA interstitial I_MA_^0^ creates one donor level very close to the conduction band edge,^48,50^ implying that, at room or higher temperatures, the MA interstitial will be ionized I_MA_^+^. Indeed, a charge density analysis shows that, for I_MA_^0^, the extra electron delocalizes over the whole lattice (see the SI (note 5)), such that it does not impact the motion of the MA interstitial much.

From the fact that no migration events are observed for MA vacancies during a 2 ns simulation at the highest temperature considered here (600 K), one can estimate a lower bound for their activation energy *E*_a_. Assuming Arrhenius behavior with a prefactor similar to the other defects *D*_0_ ≈ 10^–4^ cm^2^s^–1^, gives an attempt frequency ν_0_ ≈ 0.5 THz.^45^ The number of migration events in 2 ns then drops below one for *E*_a_ ≈ 0.4 eV.

To gain atomistic insights into the diffusion behavior of these defects, trajectories are analyzed as defects migrate. Iodide defects migrate very similar to the way they do in CsPbI_3_ and CsPbBr_*x*_I_1–*x*_ with similar diffusion coefficients and activation barriers.^45,47^ Vacancies V_I_^+^ move through a kick-out mechanism with a neighboring I atom rotating 90° around the Pb–I bond filling in the vacancy. The migration path for I_I_^–^ interstitials consists of hopping moves of an I atom from one Pb–II–Pb double bridge to a neighboring Pb–I–Pb bond to form a double bridge there.

At the elevated temperatures studied here, the MA molecules in the MAPbI_3_ lattice reorient very rapidly.^33,67–70^ To illustrate the orientation of the MA molecules as I_MA_ migrates, the angles between the C–N axis of the migrating MA molecule and that of the MA molecule in the Pb–I cage it migrates from, and that of the MA molecule in the Pb–I cage it migrates to are computed; see the SI (note 6) for details. The MA interstitial migrates through the collective reorientation of neighboring MA ions (Figure 3). At the start of the migration event, the migrating MA molecule is aligned roughly parallel to the neighboring MA molecule in the cage it is migrating to (Figure 3a). As the migrating molecule enters that cage, it pushes the molecule that is already there to a perpendicular orientation (Figure 3b) before the two end up in a roughly parallel orientation in that cage (Figure 3c).

**Figure 3:**
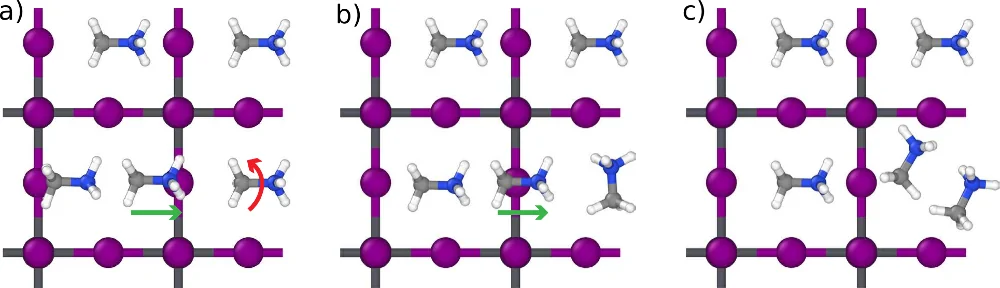
Schematic representation of the diffusion of the MA interstitial. Labels a), b), and c) represent the initial, intermediate and final state, respectively. The red arrows represent the rotation of the MA ion along the C–N axis, and the green arrows represent the migration paths.

To place our results in context, we compare the calculated migration barriers (Table 2) with the wide range of experimentally reported activation energies (Table 3) for ion migration in MAPbI_3_. Most experimental data gathered on ion migration in MAPbI_3_ have been assigned to halide migration,^71^ with reported activation energies from 0.10 to 0.68 eV, extracted with different measurement techniques, including conductivity/current measurements,^16,19,22,23^ capacitance/impedance measurements,^24,28–31^ photoluminescence,^14,20,21^ photocurrent relaxation,^15^ current response measurements,^18^ ionic current measurements,^25^ and transient ion drift.^26^ Some experiments also report results assigned to the diffusion of MA cations with barriers between 0.23 and 0.94 eV, from conductivity/current measurements,^17,23^ impedance measurements,^27,28^ deep-level transient spectroscopy,^30^ and ionic current measurements.^25^ Apart from technical issues and interpretational difficulties regarding the experimental data,^71,72^ it is clear that there is a considerable spread in these numbers.

**Table 3:** Experimentally Reported Activation Energies for Defect Migration

Technique	Activation energy (eV)	Migrating Species
Bulk conductivity^16^	0.43	–
Conductivity^17^	0.36	MA^+^ ion
Current density^19^	0.23–0.31	I_I_^–^
Conductivity^22^	0.08–0.14 and 0.47	–
Current density^23^	0.10 and 0.41	V_I_^+^ and V_MA_^–^
Ionic current^25^	0.28 and 0.94	V_I_^+^ and V_MA_^–^
Current response^18^	0.10–0.18	V_I_^+^
Impedance spectroscopy^27^	0.58	MA^+^ ion
Impedance spectroscopy^28^	0.55 and 0.68	V_I_^+^ and V_MA_^–^
Impedance spectroscopy^29^	0.13	halide ions
Impedance spectroscopy^30^	0.18	I_MA_^+^
Impedance spectroscopy^31^	0.50	halide anions
Capacitive signature^24^	0.36	–
Deep-level transient spectroscopy^30^	0.23 and 0.14	I_MA_^+^ and I_I_^–^
Photoluminescence rise rates^14^	0.27	halide ions
Photoluminescence rise rates^20^	0.14	Iodide defects
Photoluminescence rise rates^21^	0.19	Iodide defects
Photocurrent relaxation^15^	0.60–0.68	V_I_^+^
Transient ion-drift^26^	0.29 and 0.39–0.90	I^–^ ion and MA^+^ ion

Calculated activation barriers are typically based on NEB calculations, with numbers reported for the iodide vacancy (0.08–0.58 eV), the iodide interstitial (0.08 eV), and the MA vacancy (0.46–0.96 eV).^15,34–37,39,73^ The considerable spread in those numbers is associated with the difficulties of finding the lowest energy path for diffusion in a soft material such as MAPbI_3_, where moreover the asymmetry of the MA cation may give rise to multiple competing paths.

Our MD simulations sample all these paths according to the appropriate thermal distribution, and yield a consistent set of activation energies in a narrow range of 0.15–0.20 eV, thereby supporting the lower values of the experimental spread. One should remark that, in order to gather sufficient statistics regarding migration events, we have performed the simulations at elevated temperatures, *T* ≥ 500 K, and assume that the Arrhenius expression for the diffusion coefficient, eq 2, holds for temperatures down to room temperature. This assumption might in principle be tested using rare-event sampling techniques, but that would involve a considerable computational effort outside the scope of the present paper.

As for the identification of the moving halide defect, from the simulations there is little difference in the mobility of the iodide vacancy or interstitial. Under equilibrium conditions the interstitial is the most favored point defect,^48,50^ but under nonequilibrium conditions, the vacancy might of course occur.

As for the MA defect, there is a clear distinction between interstitial and vacancy, where the simulation show no migration events for the MA vacancy within the simulated temperature and time scales, whereas the MA interstitial has a mobility comparable to that of the iodide defects. The latter challenges the common assumption that MA migration is intrinsically slow due to its molecular nature. Our calculated migration barrier of 0.20 eV for I_MA_^+^ (Table 2) is in agreement with the values obtained experimentally by Reichert et al. (Table 3) using impedance spectroscopy (0.18 eV) and deep-level transient spectroscopy (0.23 eV).^30^ Again, equilibrium conditions favor the formation of interstitials,^48,50^ with the MA vacancy only occurring under very iodine-rich conditions.

Some of the spread in experimental numbers might be explained by a difference in time and length scales caused by the inhomogeneity of the samples. These are typically multicrystalline thin films, where diffusion in a crystalline grain will be different from that at grain boundaries/surfaces or between grains. As our simulations only consider bulk diffusion, this might suggest that the higher numbers observed in experiments refer to intergrain diffusion.

In summary, we study the diffusion of iodide and MA interstitials and vacancies in MAPbI_3_. We trained a neural network potential for the most stable charge states of these defects (I_I_^–^, I_MA_^+^, V_I_^+^, and V_MA_^–^), and separate neural network potentials for neutral MA interstitial (I_MA_^0^) and vacancy (V_MA_^0^) using DFT calculations. Using these force fields, we performed MD simulations to study the migration behavior of these defects.

We find that all defects besides MA vacancies are mobile with migration barriers in a narrow range of 0.15–0.20 eV, consistent with the lower end of experimentally reported activation energies. The migration rates of the MA interstitials are very similar to those of iodide point defects, despite MA interstitials being much bulkier than iodide defects. We attribute this finding to the collective reorientation of neighboring MA ions. In addition, our findings suggest that unlike iodide point defects, where the migration rates depend on the defect charge state, those of MA defects are not impacted by the charge state.

These findings provide a unified microscopic interpretation of ion migration in MAPbI_3_ and help reconcile the wide range of activation energies reported experimentally. We show that low activation energies reflect intrinsic bulk diffusion dominated by iodide defects and MA interstitials, whereas higher activation energies reported experimentally likely reflect additional contributions, such as intergrain transport or formation of complex defects. Overall, our results revise the conventional picture of ionic transport in hybrid perovskites and highlight the importance of collective molecular motion in enabling fast ion migration.

## Supplementary Material



## Data Availability

The training sets for the models used in this article are available in ref 74.

## Notes

MAPbI_3_ Models training parameters, validation; geometries along NEB paths; molecular dynamics simulations details; I_MA_^+^ charge density analysis; MA orientation during I_MA_ migration (PDF)
