# Supplementary material for: A Unified Microscopic Picture of Cation and Anion Migration in MAPbI3
Source: J Phys Chem Lett. 2026 Oct 15;17(43):12234–40. doi: 10.1021/acs.jpclett.6c02367 (PMC13726468; doi:10.1021/acs.jpclett.6c02367)
Supplement: Supplementary file 1 [file jz6c02367_si_001.pdf]

Supporting Information: A unified microscopic picture of cation and anion migration in $MAPbI_3$

Viren Tyagi,[†] Geert Brocks,[†,‡] and Shuxia Tao[*,†]

†*Department of Applied Physics and Science Education, Eindhoven University of Technology, 5600 MB, Eindhoven, The Netherlands*

‡*Computational Chemical Physics, Faculty of Science and Technology and MESA+ Institute for Nanotechnology, University of Twente, 7500 AE, Enschede, The Netherlands*

E-mail: s.x.tao@tue.nl

Contents

1 Model training **S3**

2 Model validation **S6**

2.1 NEB parameters and geometries S6

2.2 Forces, energies and stress tensors S9

2.3 Energy drift S17

3 $MAPbI_3$ phase transition **S19**

4 Production runs and diffusion coefficients **S22**

5 Charge density analysis **S25**

6 MA orientation during I_{MA} migration **S26**

References **S28**

1 Model training

Training structures are sampled from three constant temperature MD runs using the on-the-fly learning procedure as implemented in VASP,[1–5] for both neutral and positively charged methylammonium interstitial (I_{MA}^{0} and I_{MA}^{+}), neutral and negatively charged methylammonium vacancy (V_{MA}^{0} and V_{MA}^{-}), and negatively charged iodide interstitial (I_{I}^{-}) and positively charged iodide vacancy (V_{I}^{+}). These runs are performed for $105\,\mathrm{ps}$ with an MD timestep of $3\,\mathrm{fs}$ and the mass of H increased to $4\,\mathrm{amu}$ to increase sampling, using $2 \times 2 \times 2$ cubic supercells of $MAPbI_3$ (96 atoms) with one MA or I point defect in them. Three such runs are performed for each system at temperatures between $500\,\mathrm{K}$ and $750\,\mathrm{K}$ (Table S1) in NpT ensembles at 10^5 Pa pressure. To maintain constant temperature and pressure, Parinello-Rahman dynamics[6,7] is used with friction coefficients set to $3\,\mathrm{ps}^{-1}$ for all atomic species and lattice degrees of freedom.

Table S1: The number of structures (n) in the training set and temperatures at which the training runs are performed for different defect systems.

System	n	Step 1	Step 2	Step 3
I_{MA}^{0}	1485	$600\,\mathrm{K}$	$700\,\mathrm{K}$	$500\,\mathrm{K}$
I_{MA}^{+}	1596	$700\,\mathrm{K}$	$700\,\mathrm{K}$	$500\,\mathrm{K}$
V_{MA}^{0}	1339	$600\,\mathrm{K}$	$700\,\mathrm{K}$	$500\,\mathrm{K}$
V_{MA}^{-}	1328	$600\,\mathrm{K}$	$700\,\mathrm{K}$	$500\,\mathrm{K}$
I_{I}^{-}	1818	$600\,\mathrm{K}$	$750\,\mathrm{K}$	$750\,\mathrm{K}$
V_{I}^{+}	1501	$600\,\mathrm{K}$	$750\,\mathrm{K}$	$750\,\mathrm{K}$

The total number of structures sampled for all systems is given in Table S1. DFT single-point calculations are performed on all these structures in VASP. The projector-augmented wave (PAW)[8] technique is used to model electron-ion interactions, with the outermost electrons of I ($5s^25p^5$), Pb ($6s^26p^2$), H ($1s^1$), C ($2s^22p^2$), and N ($2s^22p^3$) treated as valence electrons, and applying the standard VASP PAW potentials. The electronic interactions are modeled using the SCAN+rVV10 functional,[9,10] as it has been shown to reliably describe the dynamics of the MA ion and defect formation energies.[11–13] An energy convergence criterion of $10^{-6}\,\mathrm{eV}$ is used, along with a $2 \times 2 \times 2$ Monkhorst-Pack k-point grid[14] and a kinetic energy cutoff of $500\,\mathrm{eV}$.

Three different Allegro models[15,16] are trained, one for all charged defects (I_{MA}^{+}, V_{MA}^{-}, I_{I}^{-}, and

V_I^+), one for I_{MA}^0, and one for V_{MA}^0. The defects have these charge states when the Fermi level is well inside the band gap,[13] and a single force field should be able to capture this. The neutral I_{MA}^0(V_{MA}^0) can only be found when the Fermi energy is very close or in the conduction (valence) band, and separate force fields have to be constructed to describe these situations.

All of these models are trained using a radial cutoff of $6.5\,\text{Å}$ with interatomic distances projected onto a radial basis using trainable Bessel functions, and 2 tensor product layers. 32 ordered-pair tensor features are used, expanded using spherical harmonics with a maximum angular quantum number (L_{max}) of 2, while preserving full $O(3)$ symmetry. The 2-body latent multilayer perceptron (MLP) had the dimensions [128, 256, 512, 1024] and SiLU nonlinearity, and the latent MLP had the dimensions [1024, 1024, 1024] also with SiLU nonlinearity. To predict the pair energies, the final MLP has dimensions [128] without nonlinearity.

For all three models, the training and the validation sets are divided into 80% and 20% of the total number of structures (Table S2), respectively, which are shuffled at each epoch during training. The training is performed using the total energy of the system, forces acting on the atoms, and stress tensors. The per-atom MSE loss function is used, with the weight set to 1 for both energy and forces. The Adam optimizer in PyTorch is used with the default parameters $\beta_1 = 0.9$, $\beta_2 = 0.99$, and $\epsilon = 10^{-8}$. The learning rate of 0.001 and the batch size of 5 are used. The number of epochs the training of each model ran for is given in Table S2.

The root mean squared error (RMSE) in energy and forces during training and validation is given in Figure S1. The total number of epochs, and training and validation errors in energy and forces for the final model are given in Table S2.

Table S2: The total number of structures in the training and validation sets, number of epochs, training and validation errors in energy and forces for all models.

Model	n	epochs	F_{RMSE}(meV/Å)		E_{RMSE}(meV/atom)	
			Training	Validation	Training	Validation
charged defects	6243	970	6.5	17.9	1.09	1.09
I_{MA}^0	1485	850	5.8	24.1	0.41	0.45
V_{MA}^0	1339	954	5.2	20.9	0.40	0.43

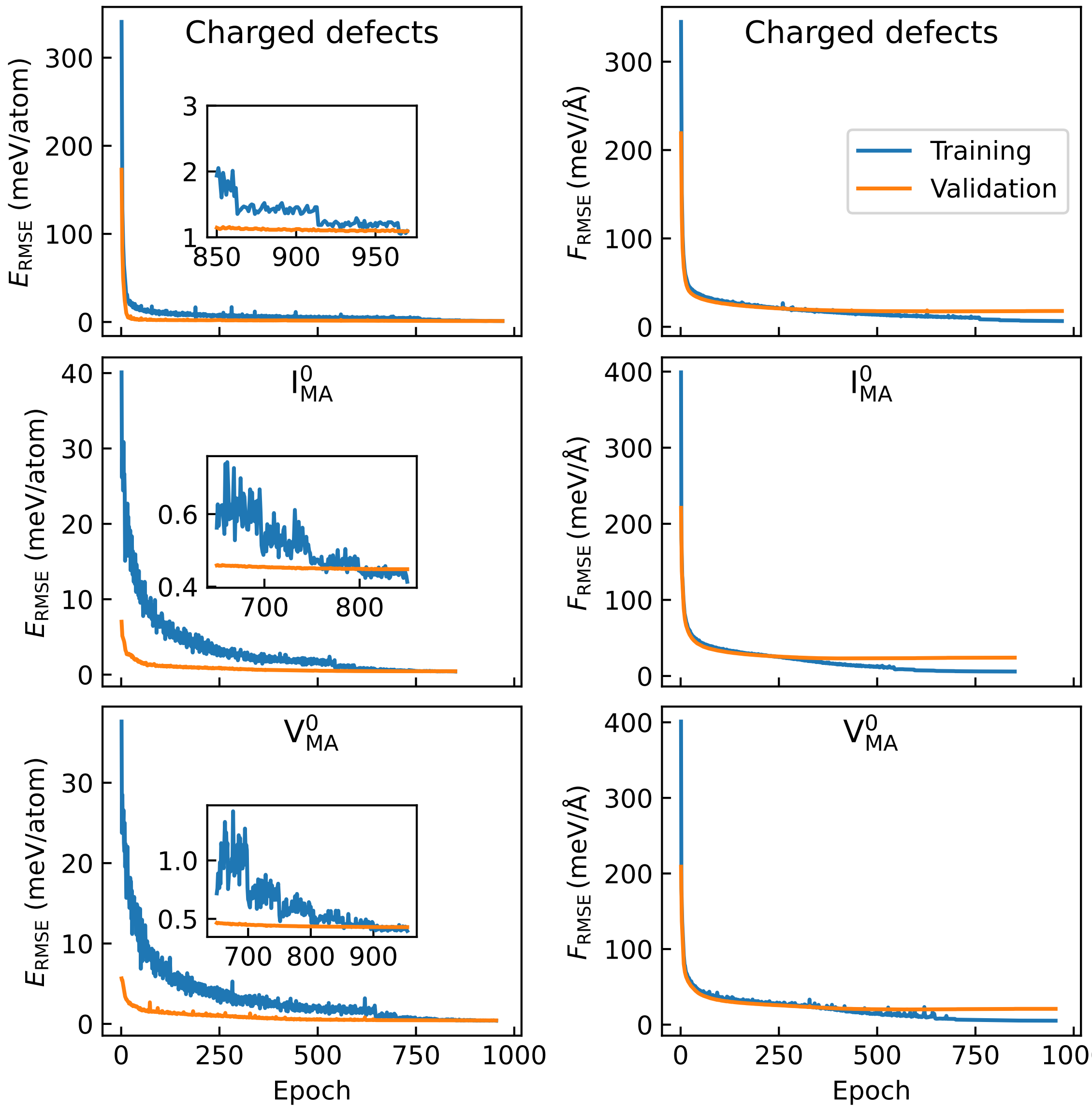

Figure S1: Root mean squared errors in energy (E_{RMSE}) and forces (F_{RMSE}) during model training and model validation of all models.

2 Model validation

2.1 NEB parameters and geometries

The energies calculated with the NNPs are compared with those calculated using DFT for paths constructed using the climbing image nudged elastic band (CI-NEB) method.[17] These paths are constructed using $2 \times 2 \times 1$ supercell of $MAPbI_3$ (16 units) with one MA or I point defect. Three intermediate images, connected by springs with spring constants $5\,\mathrm{eV}/\mathrm{\AA}^2$, are optimized using the same DFT parameters as in Section 1, and a $2 \times 2 \times 3$ Monkhorst-Pack k-grid. The geometries of the starting, saddle and final points of the CI-NEB paths are given in Figure S2- S7 for all defects. The geometries are visualized in VESTA[18]

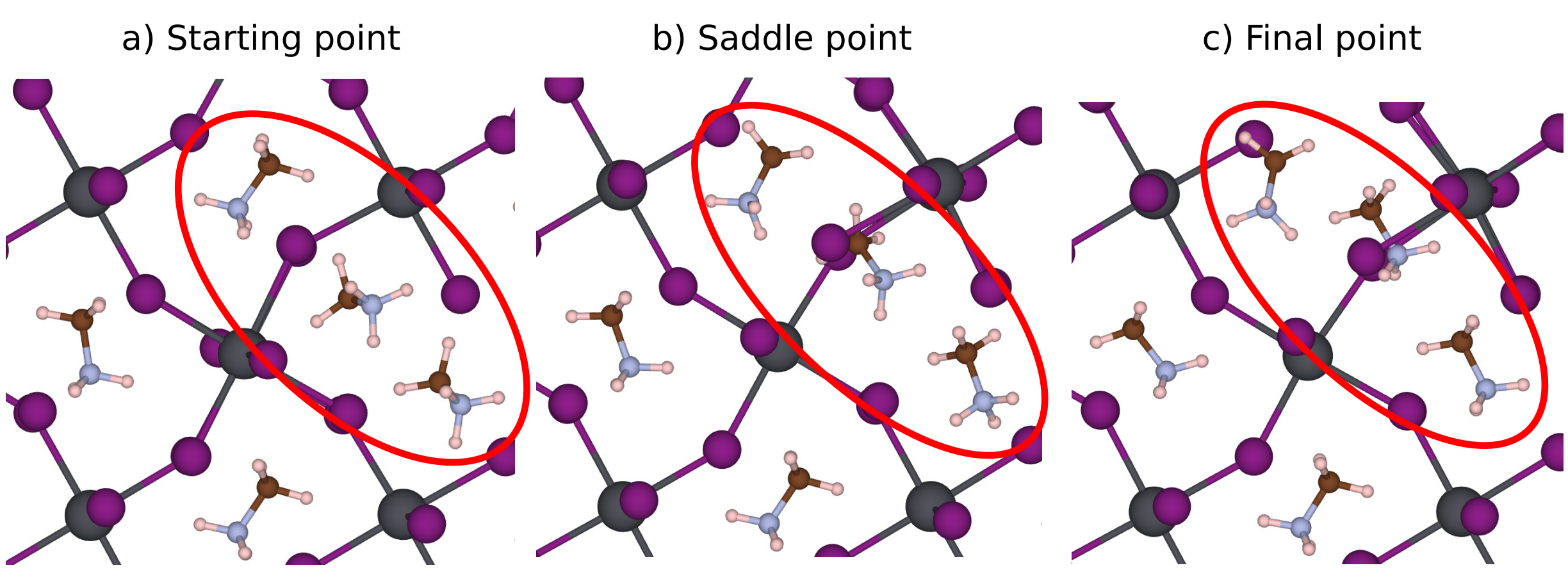

Figure S2: The geometries of the starting, saddle and final points along the CI-NEB path for I_{MA}^{+}. The defect environment is indicated by the red ellipse.

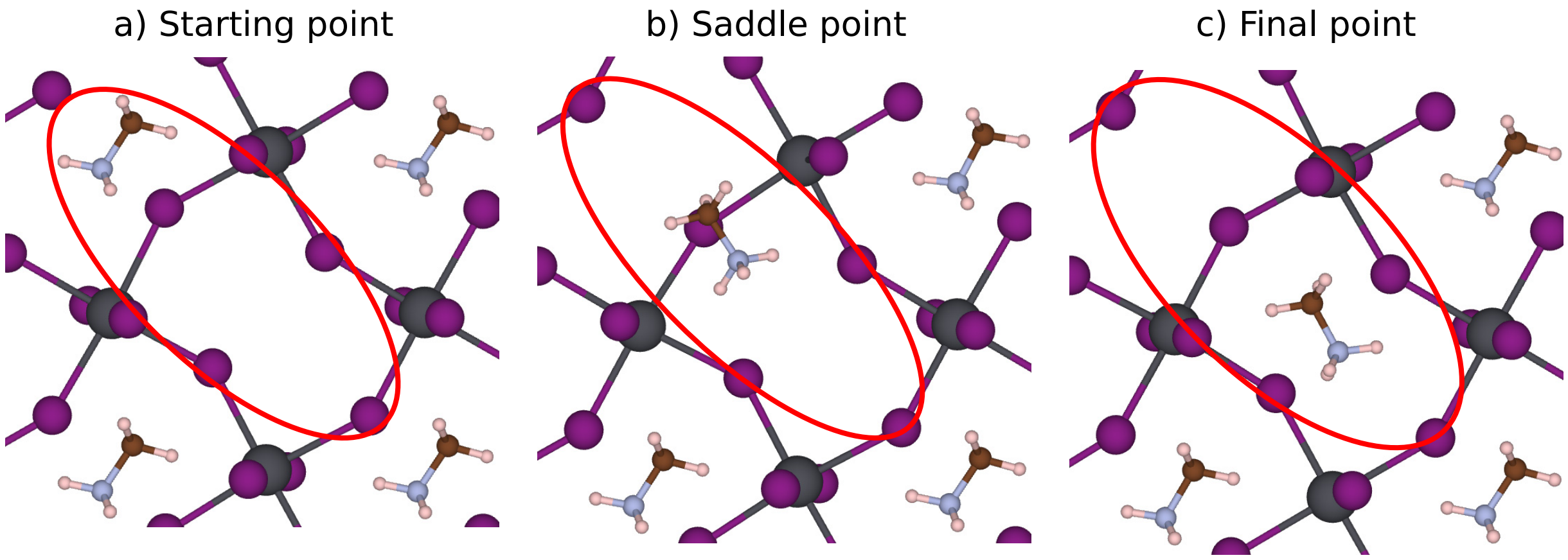

Figure S3: The geometries of the starting, saddle and final points along the CI-NEB path for V_{MA}^{-}. The defect environment is indicated by the red ellipse.

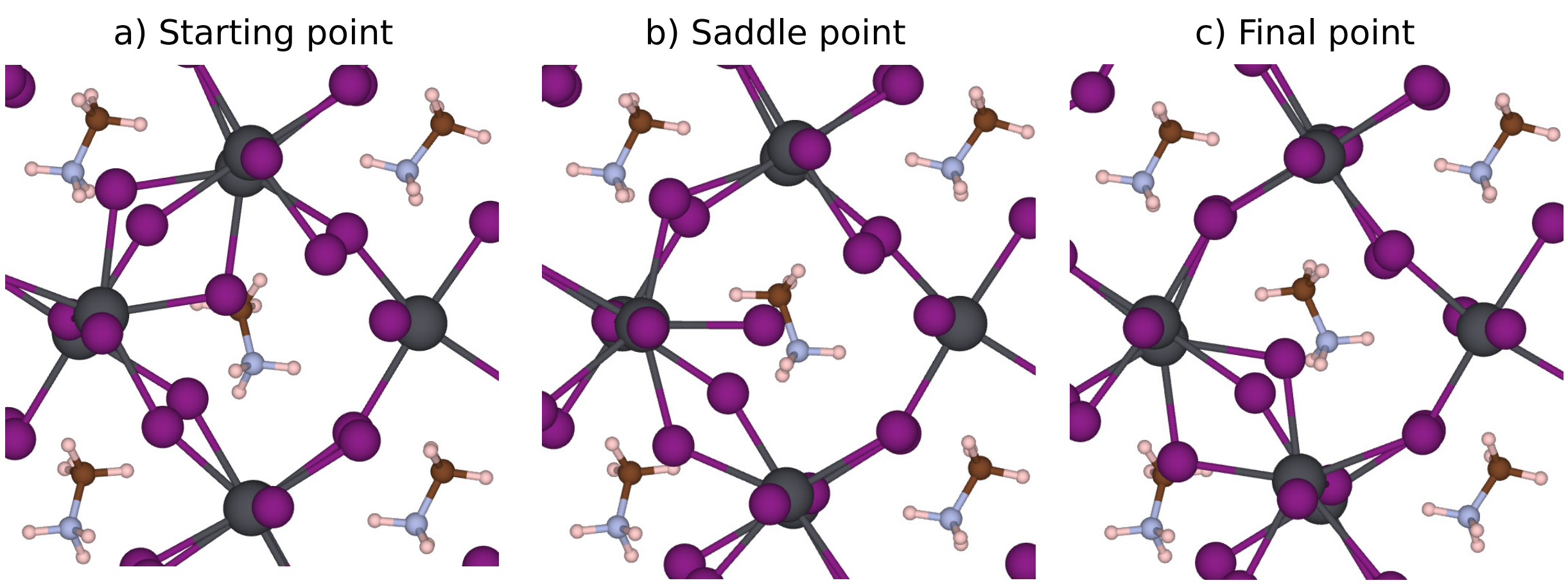

Figure S4: The geometries of the starting, saddle and final points along the CI-NEB path for I_{I}^{-}.

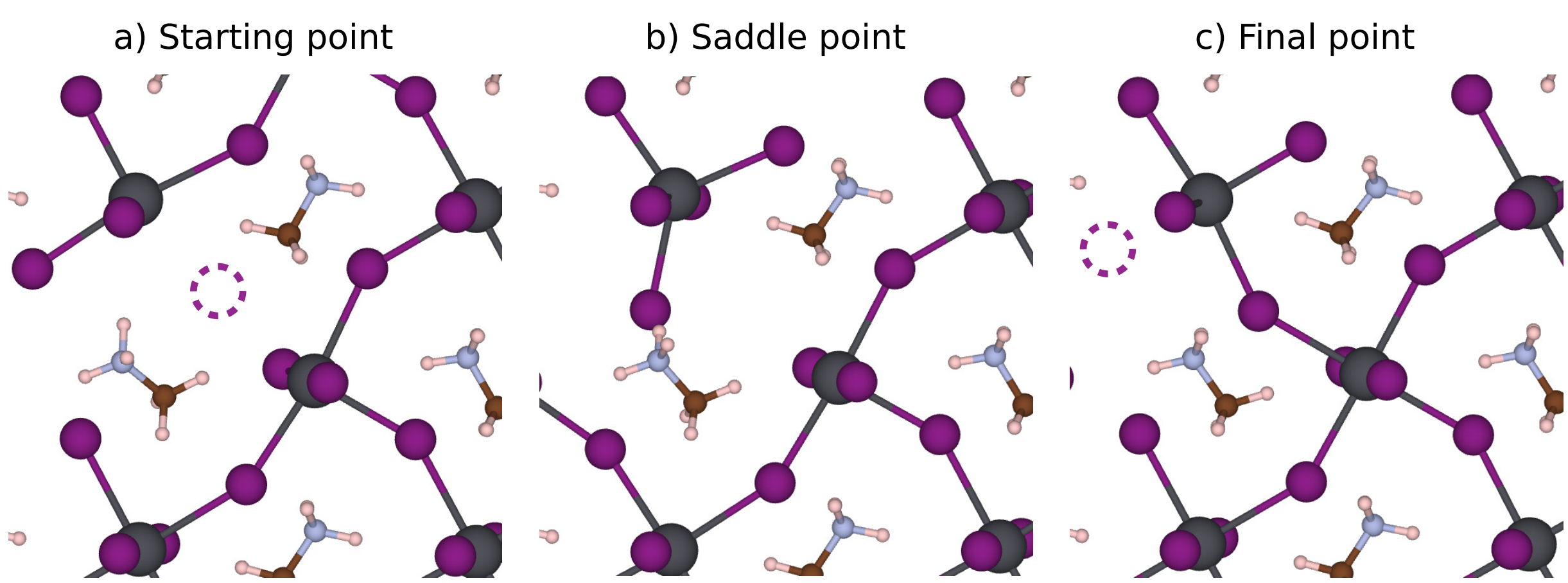

Figure S5: The geometries of the starting, saddle and final points along the CI-NEB path for V_{I}^{+}.

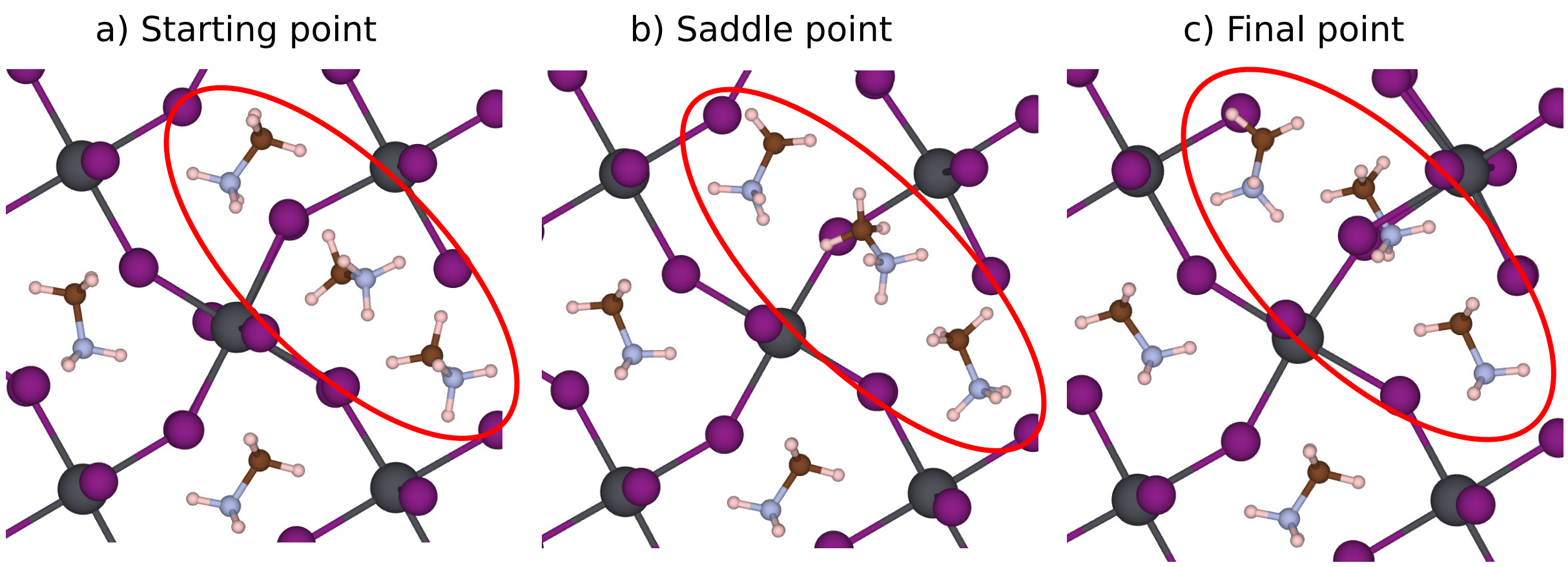

Figure S6: The geometries of the starting, saddle and final points along the CI-NEB path for I^0_{MA}. The defect environment is indicated by the red ellipse.

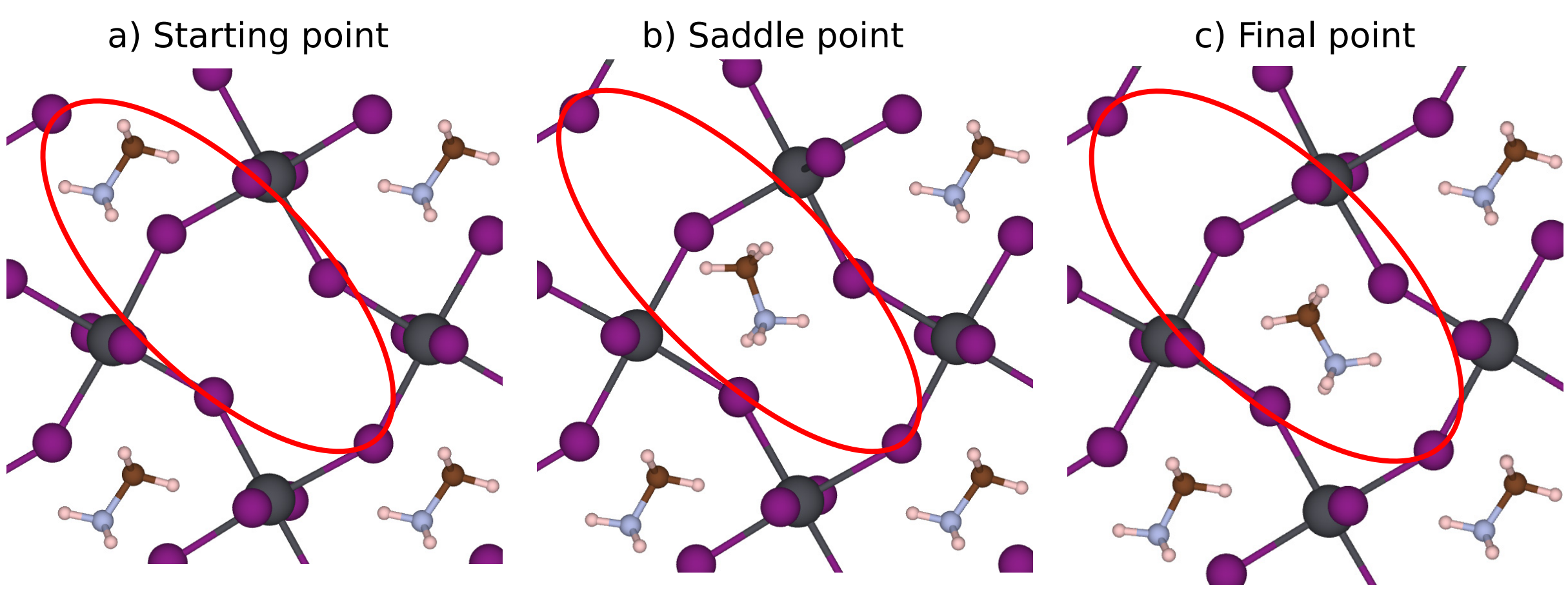

Figure S7: The geometries of the starting, saddle and final points along the CI-NEB path for V^0_{MA}. The defect environment is indicated by the red ellipse.

2.2 Forces, energies and stress tensors

To check the accuracy of the models in predicting forces ,total energies and stresses, at least 10 structures are sampled from $0.5\,\mathrm{ns}$ MD runs at $600\,\mathrm{K}$ performed using $6 \times 6 \times 6$ cubic supercells of $MAPbI_3$ (2592 atoms) with one MA or I point defect, and forces ,total energies and stresses calculated using DFT are compared with those calculated using the NNP for these structures. Sampling every 50 ps corresponds to roughly 10^2 cycles of vibrations of the Pb halide framework. Halide perovskite lattices are very soft, leading to very anharmonic motions and extremely small phonon lifetimes $\tau \sim 1/\omega$,[19] which implies that all motional correlation is lost with the sampling interval.

The forces categorized according to the atomic species acting on all atoms, with the corresponding R^2 values and mean absolute errors (MAE) for the charged defects are given in Figure S8, and for neutral defects are given in Figure S9. From these comparisons, we note that the $R^2 = 0.99$ and MAE $\leq 29.83\,\mathrm{meV/Å}$ for all systems. Considering that the forces acting on these atoms are of the order of $1\,\mathrm{eV/Å}$, the NNPs are highly accurate in calculating forces acting on all atoms.

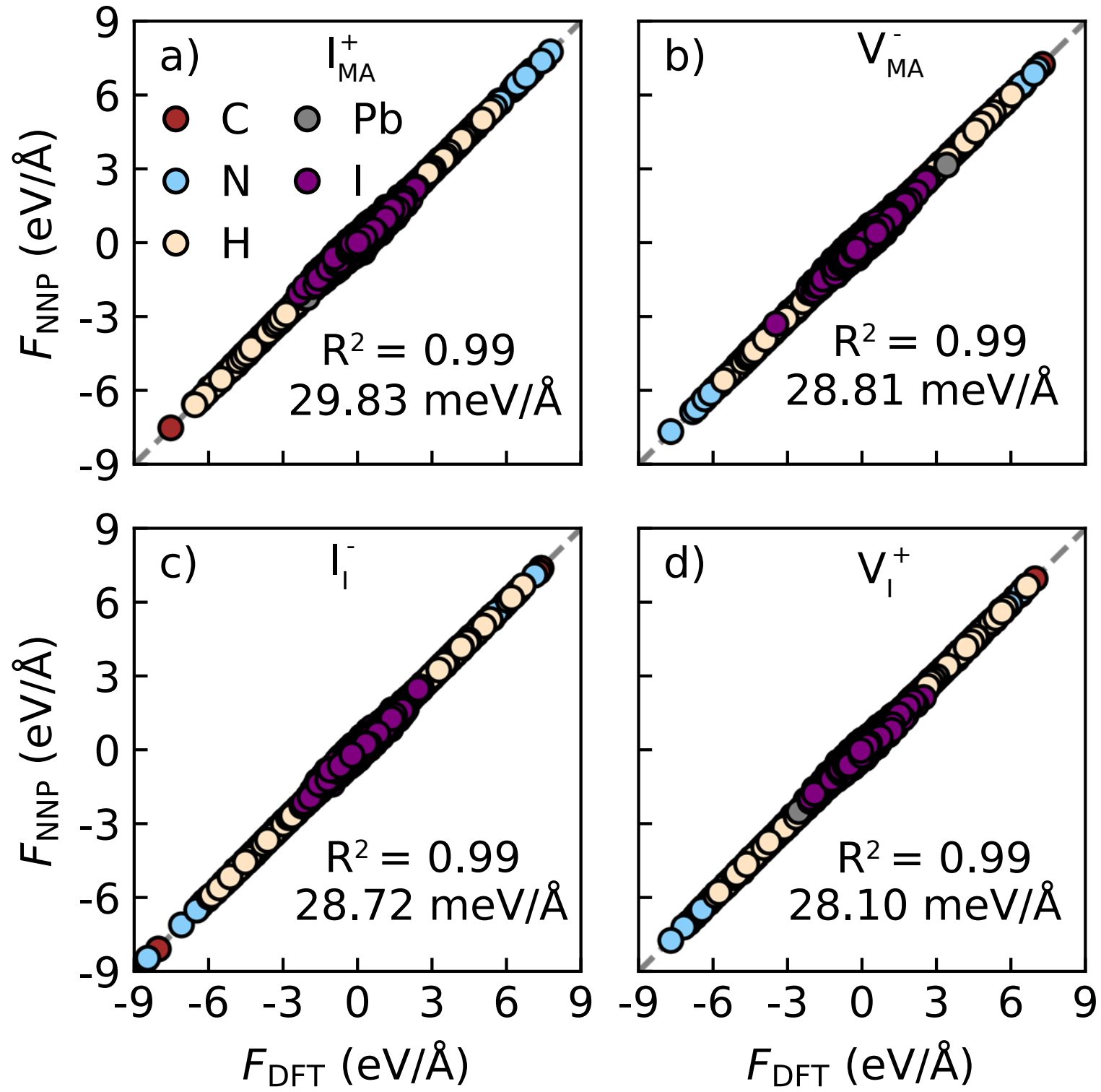

Figure S8: Comparison between forces acting on all atoms calculated by the charged defects (I_{MA}^{+},V_{MA}^{-},I_{I}^{-}, and V_{I}^{+}) NNP with forces calculated using DFT for 6 × 6 × 6 cubic supercell of $MAPbI_3$ with one MA interstitial (a), one MA vacancy (b), one I interstitial (c), or one I vacancy, along with corresponding R^2 values and mean absolute errors (MAE).

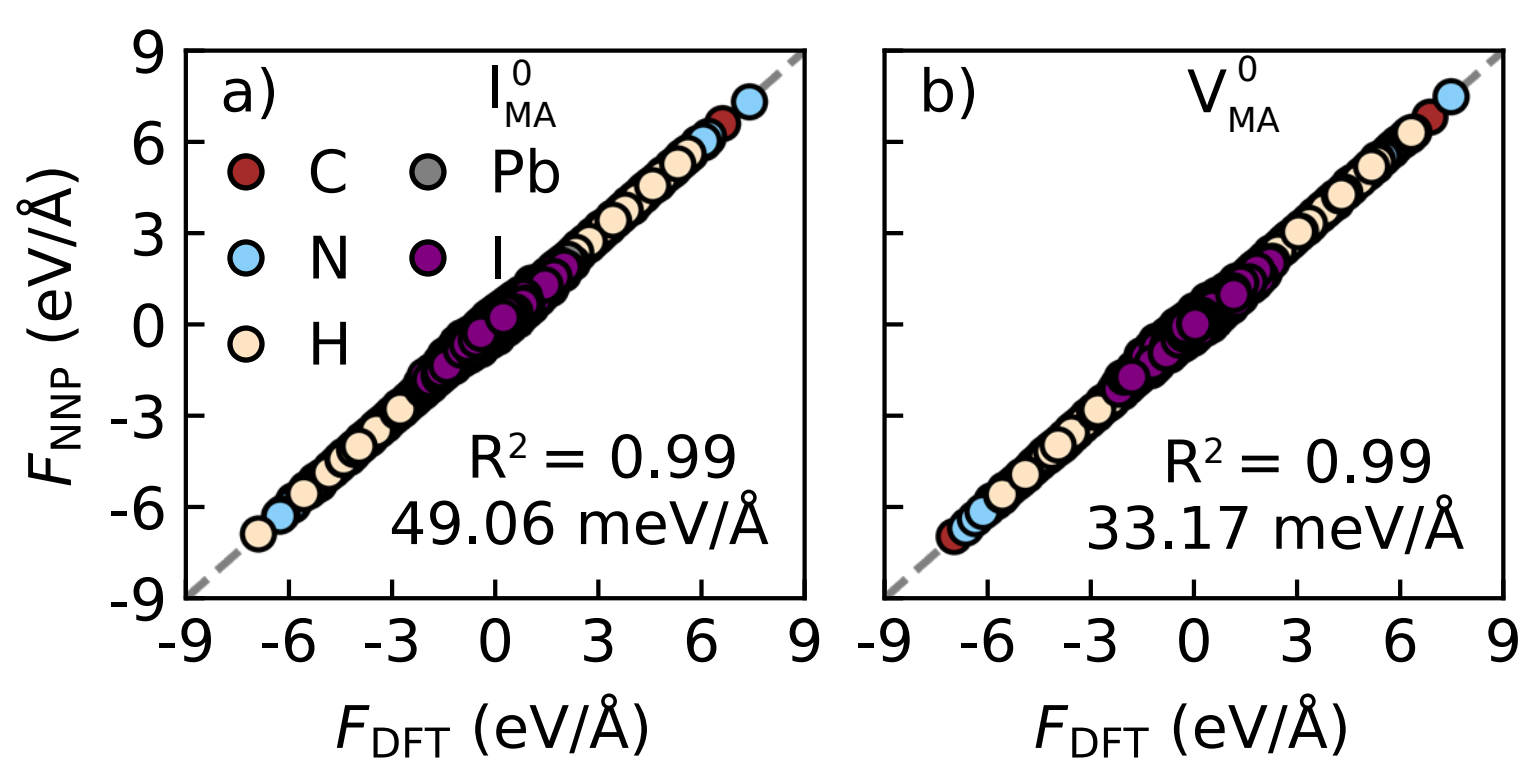

Figure S9: Comparison between forces acting on all atoms calculated by the neutral MA interstitial NNP (a) and neutral MA vacancy NNP (b) with forces calculated using DFT for 6 × 6 × 6 cubic supercell of $MAPbI_3$ with one MA point defect, along with corresponding R^2 values and mean absolute errors (MAE).

Forces acting on the atoms close to the defect are also compared. MA defect environments are identified by counting the MA neighbors of Pb, as illustrated in Figure S10. For the MA

interstitial (Figure S10a) the environment consists of Pb atoms with 9 MA neighbors, and for a MA vacancy (Figure S10b) the environment consists of Pb atoms with 7 MA neighbors. The I point defect environments are identified using the same procedure described in ref.[20] These comparisons are given in Figure S11 for charged defects, and in Figure S12 for neutral defects. From these comparisons, we conclude that the NNPs also calculate forces acting on atoms in the defect environment with high accuracy, with the $R^2 \geq 0.96$ and MAE $\leq 64.38\,\mathrm{meV/Å}$ for all systems.

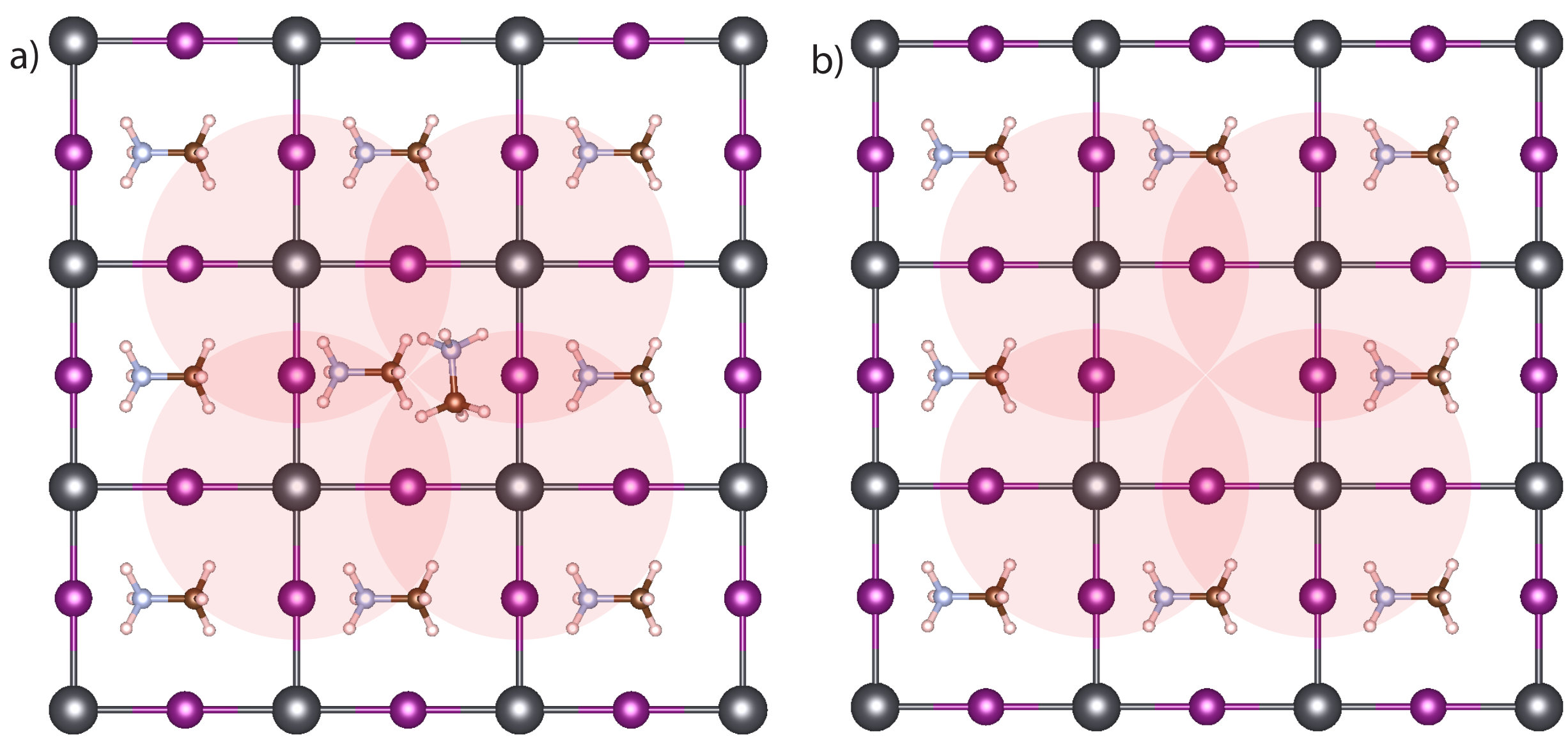

Figure S10: Illustration of how atoms close to the MA interstitial (a) and MA vacancy (b) are identified for model validation. Here, the red circles indicate the area used for selecting defect environment atoms.

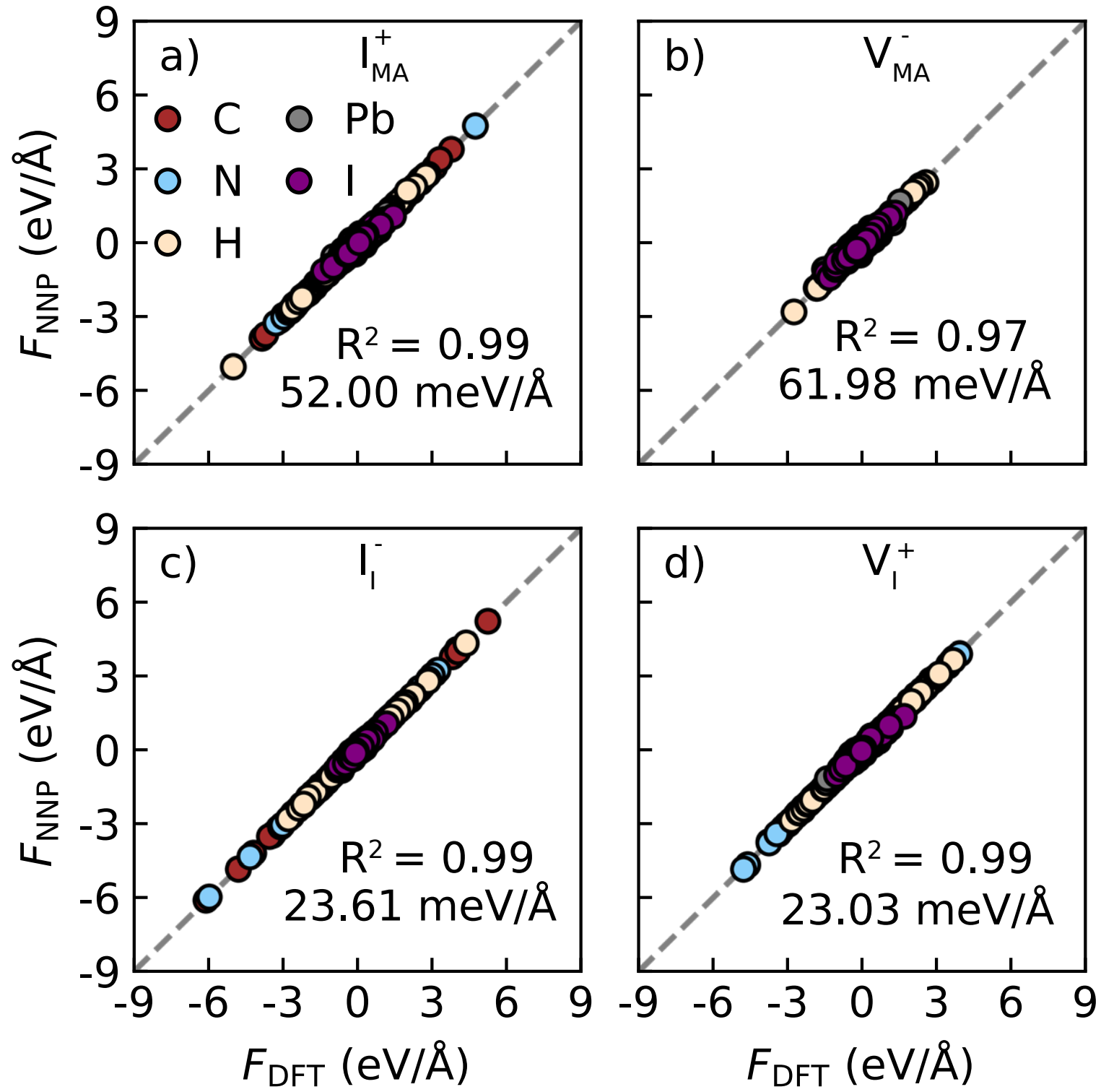

Figure S11: Comparison between forces acting on atoms in defect environment calculated by the charged defects (I^+_{MA},V^-_{MA},I^-_I, and V^+_I) NNP with forces calculated using DFT for a $6 \times 6 \times 6$ cubic supercell of $MAPbI_3$ with one MA interstitial (a), one MA vacancy (b), one I interstitial (c), or one I vacancy, along with corresponding R^2 values and mean absolute errors (MAE).

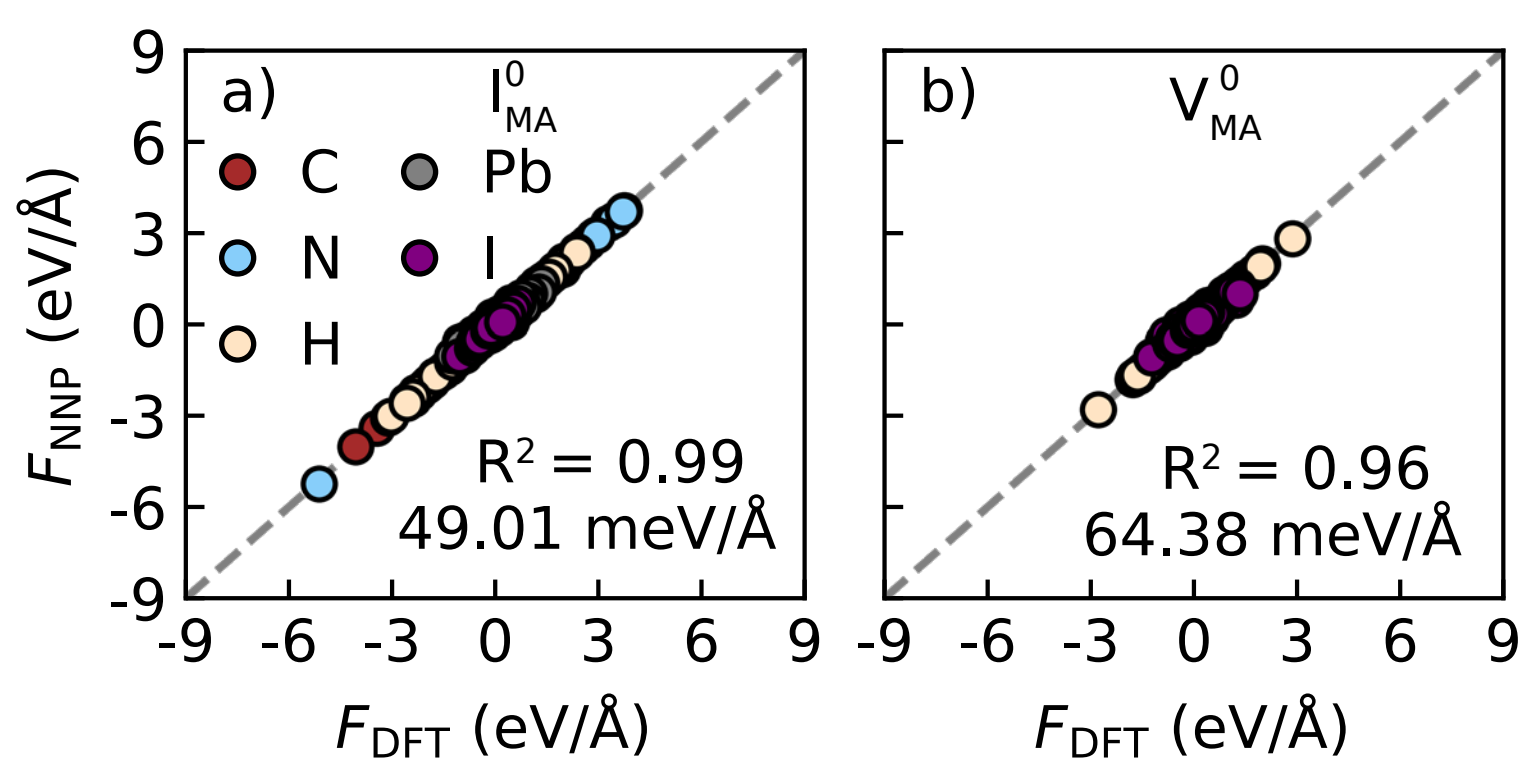

Figure S12: Comparison between forces acting on atoms in defect environment calculated by the neutral MA interstitial NNP (a) and neutral MA vacancy NNP (b) with forces calculated using DFT for $6 \times 6 \times 6$ cubic supercell of $MAPbI_3$ with one MA point defect, along with corresponding R^2 values and mean absolute errors (MAE).

The total energies along with the corresponding R^2 and MAE are given in Figure S13. From these comparisons, we note that $R^2 \geq 0.84$ and MAE $\leq 0.32\,\mathrm{meV/atom}$ for all defects.

Considering the total energies are in the range of $5\,\mathrm{meV/atom}$, the NNPs are highly accurate in calculating the total energy of the system.

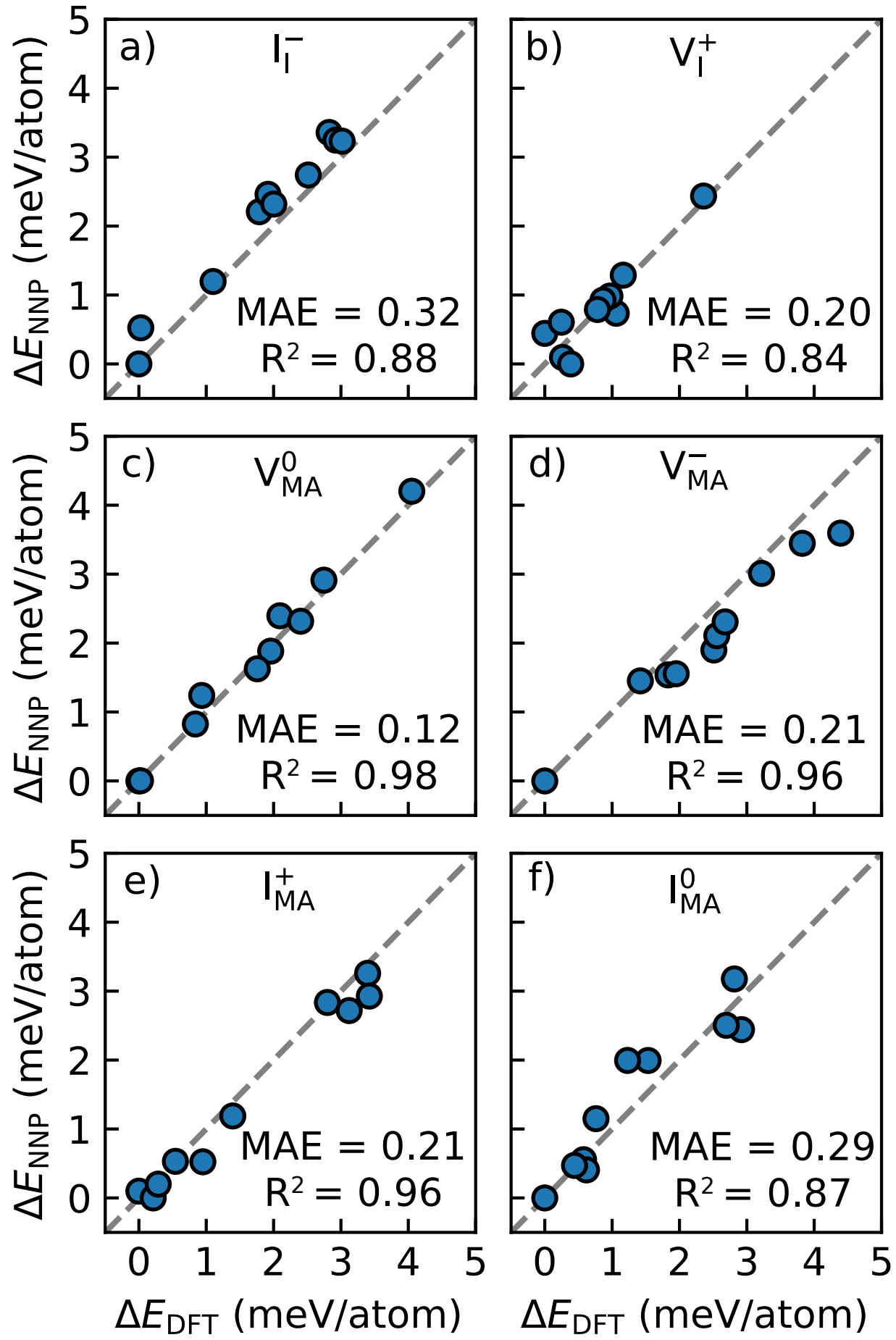

Figure S13: Comparison between the total energy of the system calculated using the NNPs with those calculated using the DFT 6 × 6 × 6 cubic supercells of $MAPbI_3$ with one a) I_I^-, b) V_I^+, c) V_{MA}^0, d) V_{MA}^-, e) I_{MA}^+ and f) I_{MA}^0, along with the corresponding R^2 values and mean absolute errors (MAE). The energy minima are set to 0.

The components of the stress tensor, along with the corresponding R^2 and MAE are given in Figure S14- S19 for all defects. From these comparisons, we note that $R^2 \geq 0.96$ and MAE $\leq 0.12\,\mathrm{kB}$ for all systems. Considering the magnitudes of the stress components are of the order of $1\,\mathrm{kB}$, we conclude that the NNPs are highly accurate in computing the stress tensors.

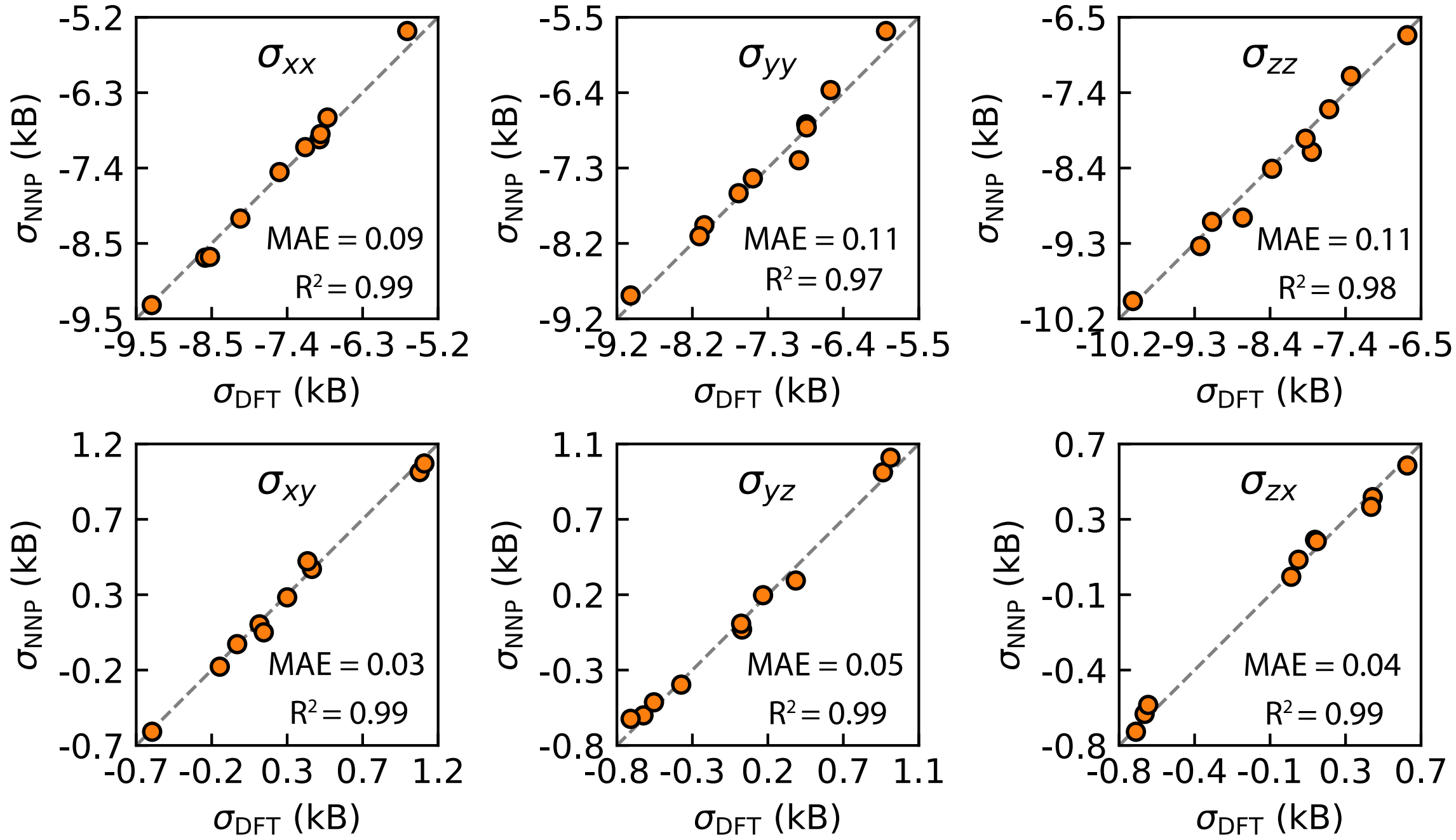

Figure S14: Comparison between the components of the stress tensor calculated by the charged defects NNP with those calculated using DFT for $6 \times 6 \times 6$ cubic supercells of $MAPbI_3$ with one I_I^-, along with corresponding R^2 values and mean absolute errors (MAE).

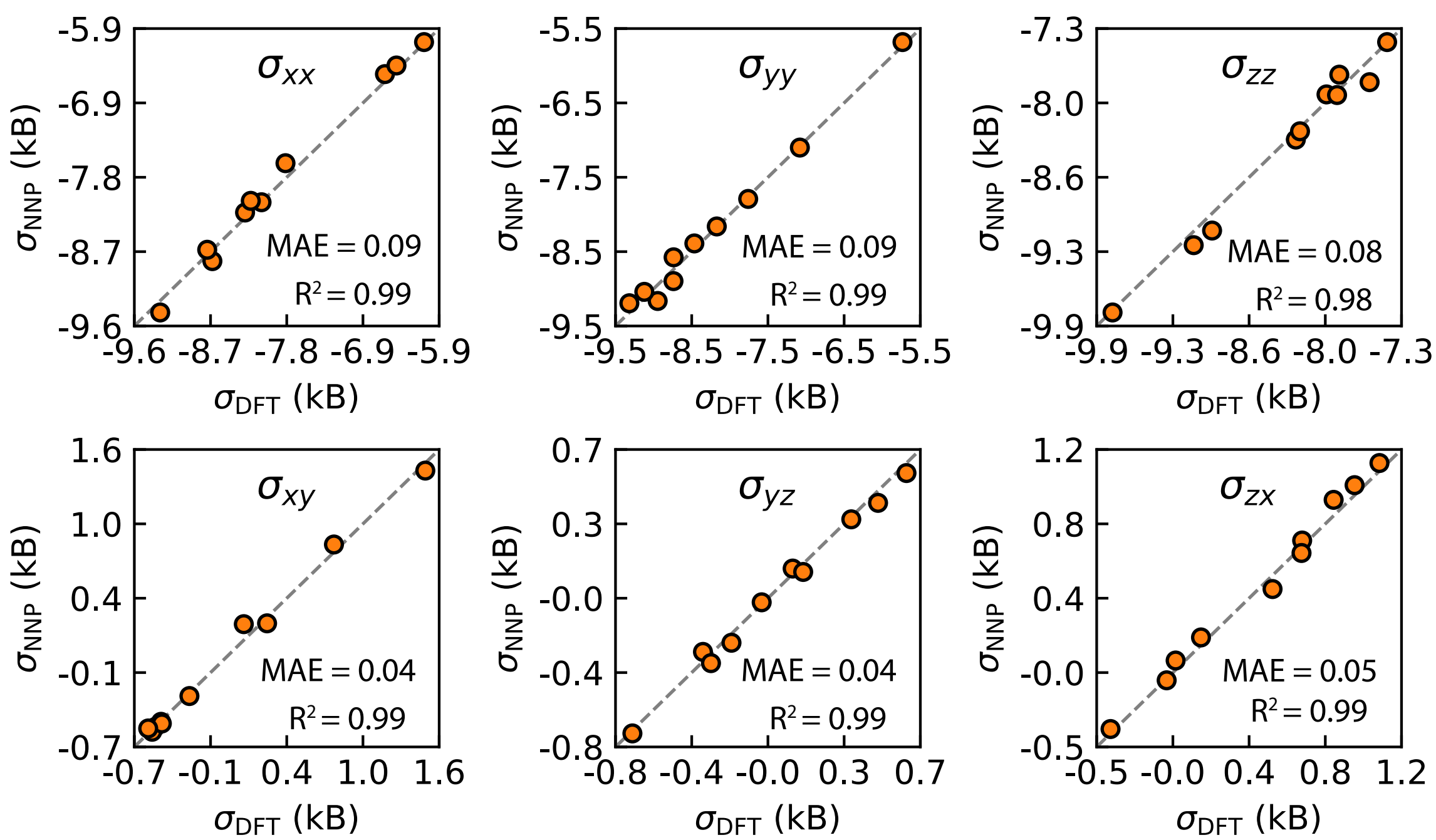

Figure S15: Comparison between the components of the stress tensor calculated by the charged defects NNP with those calculated using DFT for $6 \times 6 \times 6$ cubic supercells of $MAPbI_3$ with one I_{MA}^+, along with corresponding R^2 values and mean absolute errors (MAE).

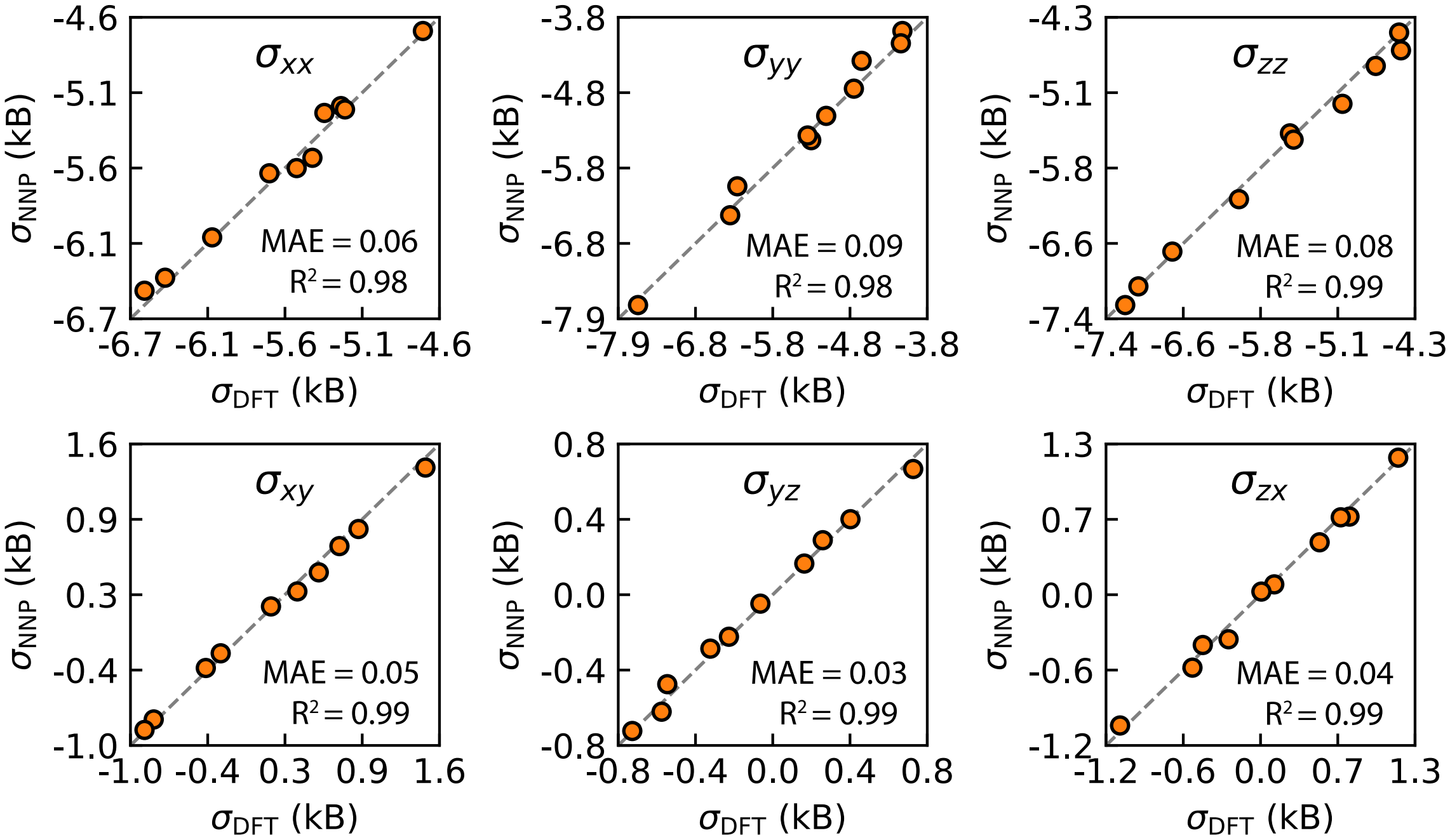

Figure S16: Comparison between the components of the stress tensor calculated by the charged defects NNP with those calculated using DFT for $6 \times 6 \times 6$ cubic supercells of $MAPbI_3$ with one V_I^+, along with corresponding R^2 values and mean absolute errors (MAE).

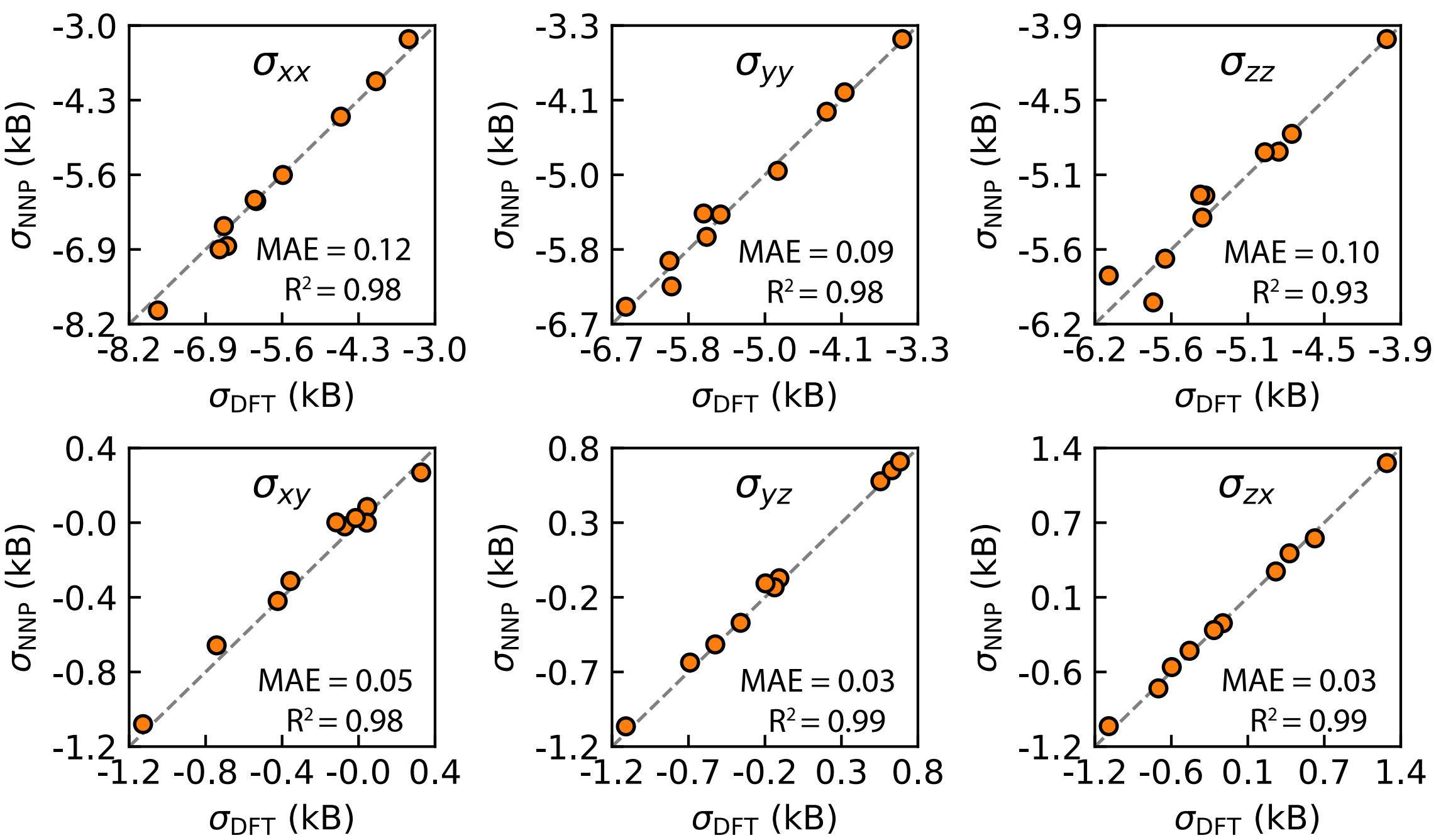

Figure S17: Comparison between the components of the stress tensor calculated by the charged defects NNP with those calculated using DFT for $6 \times 6 \times 6$ cubic supercells of $MAPbI_3$ with one V_{MA}^-, along with corresponding R^2 values and mean absolute errors (MAE).

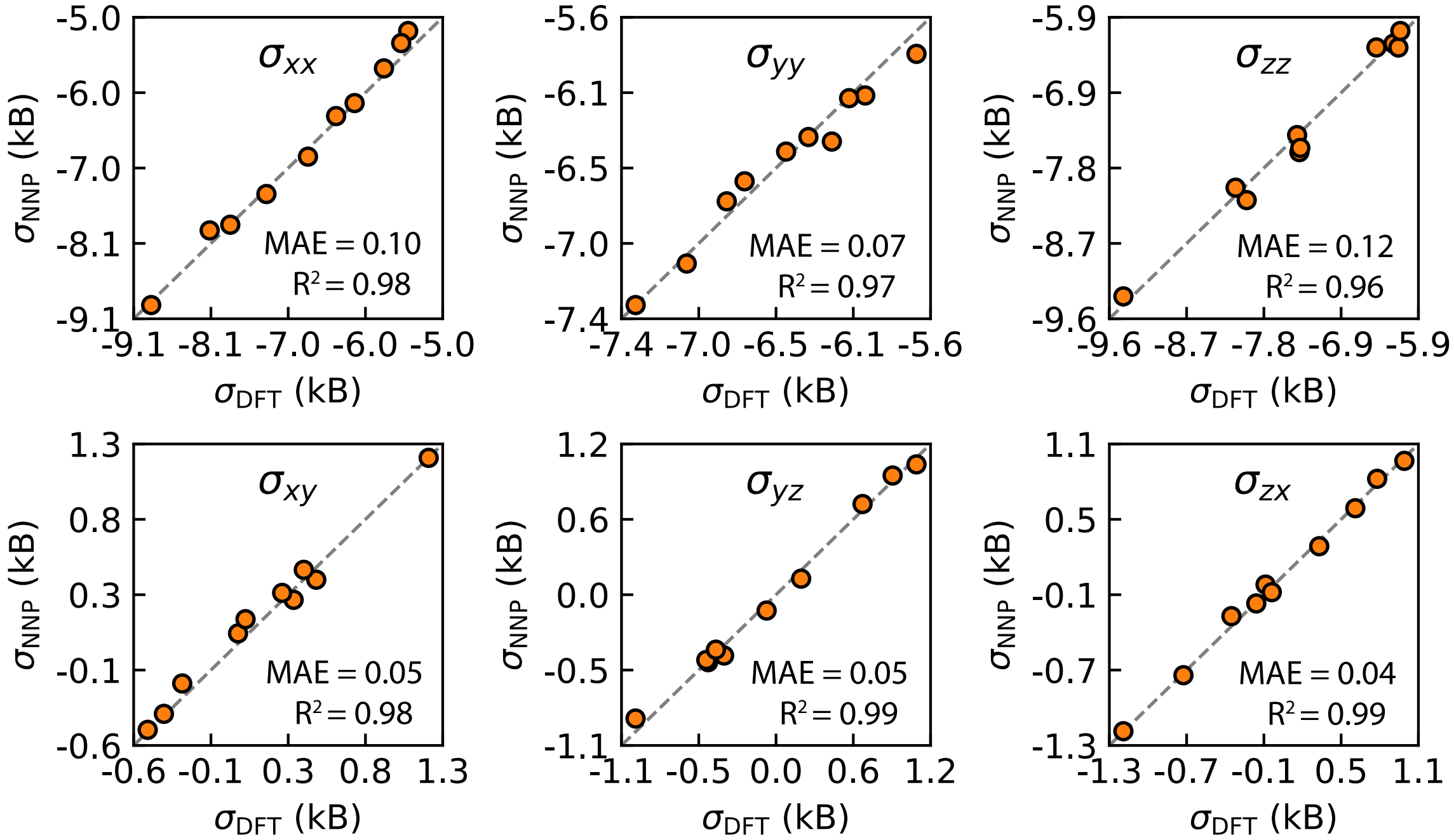

Figure S18: Comparison between the components of the stress tensor calculated by the NNP with those calculated using DFT for 6 × 6 × 6 cubic supercells of $MAPbI_3$ with one I^0_{MA}, along with corresponding R^2 values and mean absolute errors (MAE).

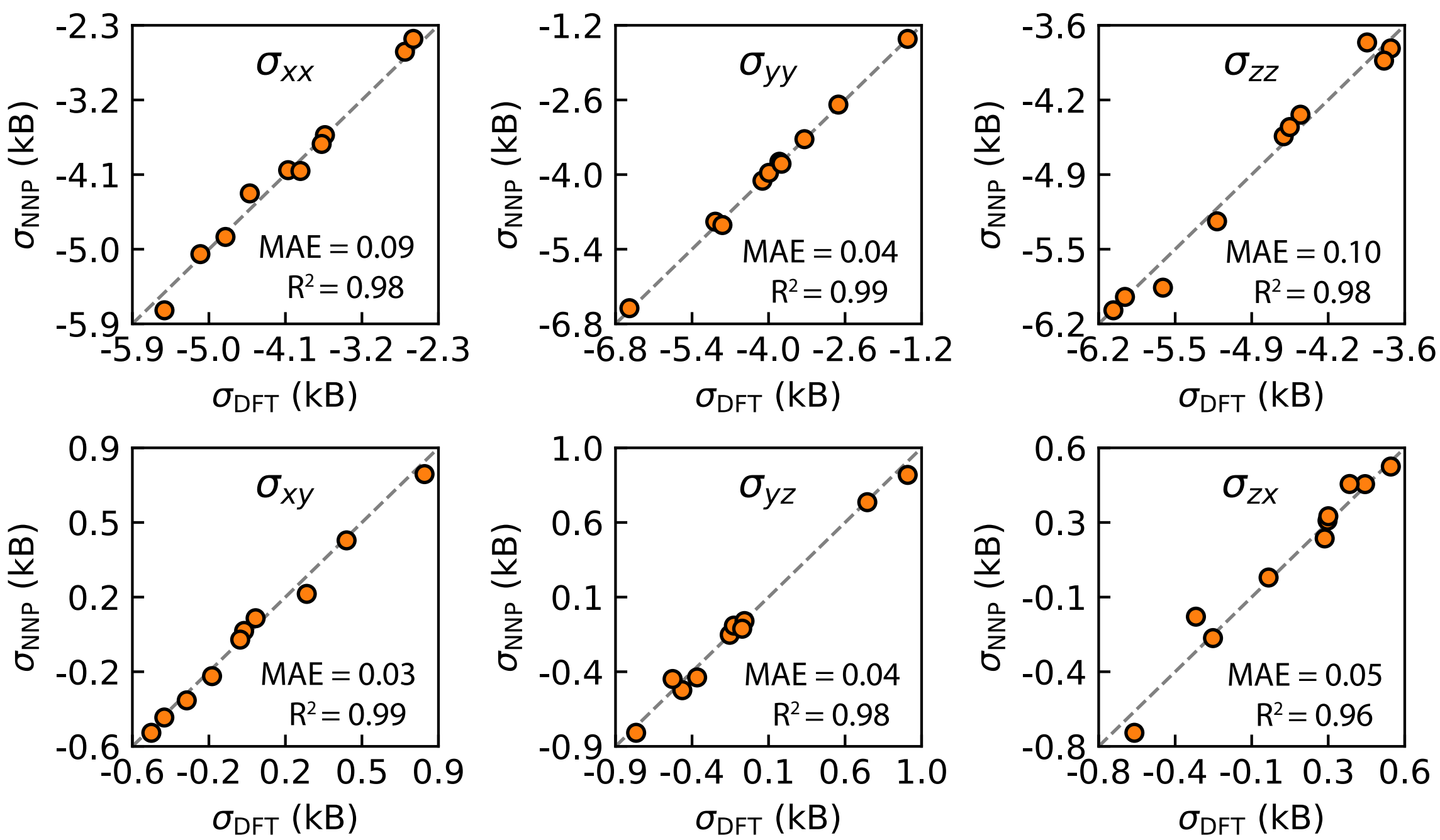

Figure S19: Comparison between the components of the stress tensor calculated by the NNP with those calculated using DFT for 6 × 6 × 6 cubic supercells of $MAPbI_3$ with one V^0_{MA}, along with corresponding R^2 values and mean absolute errors (MAE).

2.3 Energy drift

To check the stability of the force fields, the drift in the total energy of the system is computed. One $2\,\mathrm{ns}$ long run is performed with each force field in an *NVE* ensemble using $6\times6\times6$ supercells of $MAPbI_3$ (2592 atoms) with one MA point defect, MA interstitial is used for charged defects force field. The lattice constants are obtained using the procedure described in SI note 3. The structures are first equilibrated to $600\,\mathrm{K}$ for $100\,\mathrm{ps}$ with timesteps of $1\,\mathrm{fs}$ in an *NVT* ensemble using a Langevin thermostat with the relaxation time set to $0.33\,\mathrm{ps}$. The total energies of the system during the simulations, along with the respective energy drifts calculated as the slope of the linear fits, are given in Figure S20- S22. In ref,[21] the total energy drift from NVE-MD runs performed using MACE-MPO-0 is computed, and a tolerance value of $5\times10^{-4}\ \mathrm{eV/atom/ps}$ below which the energy drift is considered negligible. The energy drift observed with the models used here is at least two orders of magnitude smaller than this threshold (Figure S20- S22), hence, we conclude that the force fields are highly stable.

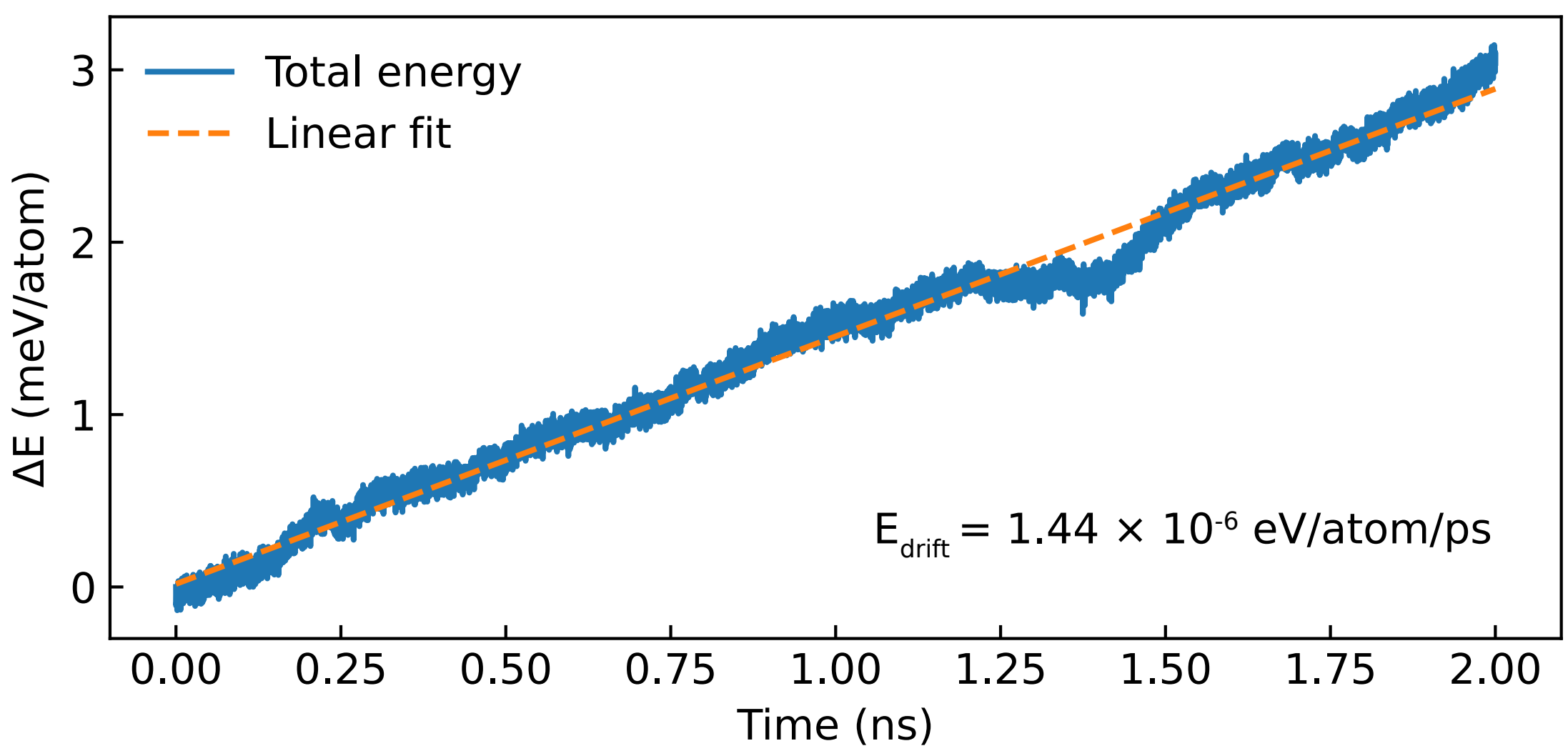

Figure S20: Total energy change (ΔE) as a function of time for a 2 ns-long NVE run using the charged defects (I^{+}_{MA},V^{-}_{MA},I^{-}_{I}, and V^{+}_{I}) force field.

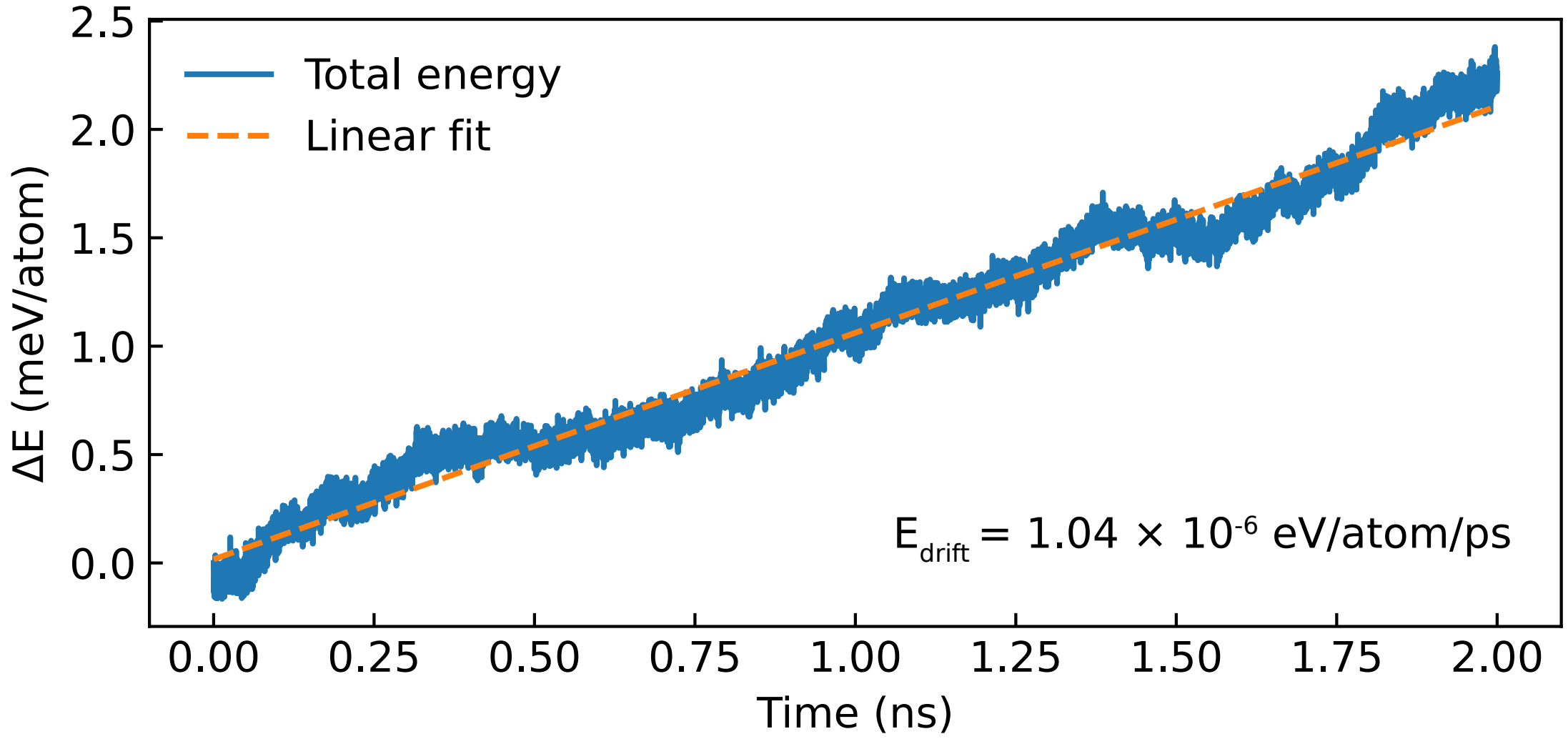

Figure S21: Total energy change (ΔE) as a function of time for a 2 ns-long NVE run using the I^0_{MA} force field.

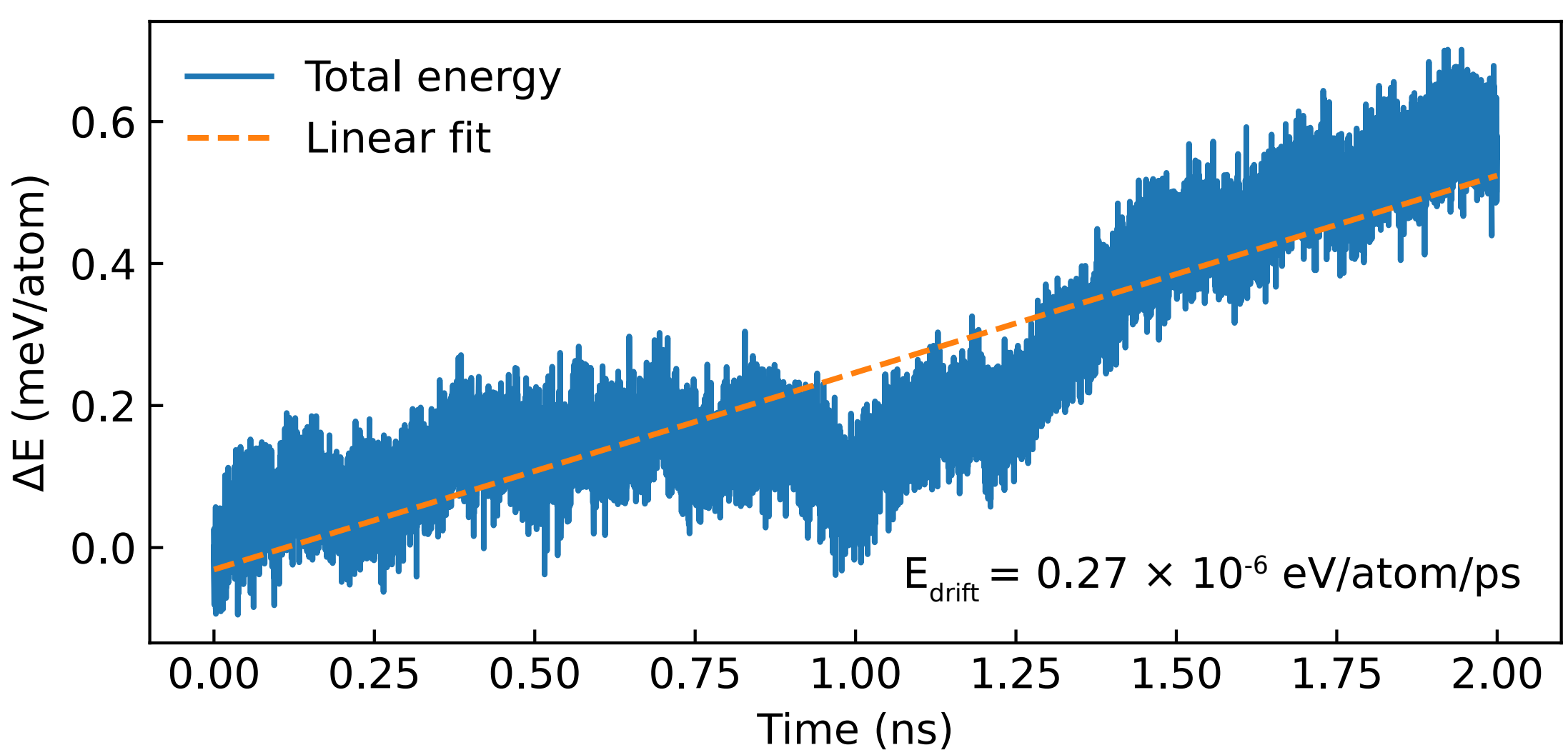

Figure S22: Total energy change (ΔE) as a function of time for a 2 ns-long NVE run using the V^0_{MA} force field.

3 $MAPbI_3$ phase transition

To test the method further and to extract the temperature dependence of the volume, a separate NNP is constructed to describe pristine $MAPbI_3$. Training structures are sampled from four constant temperature MD runs using the on-the-fly learning procedure as implemented in VASP. These runs are performed for $105\,\mathrm{ps}$ with an MD timestep of $3\,\mathrm{fs}$ and the mass of H increased to $4\,\mathrm{amu}$ to increase sampling using $2 \times 2 \times 2$ pseudo-cubic supercells of $MAPbI_3$ (96 atoms) in all three perovskite phases. The first run is performed in the cubic phase at $450\,\mathrm{K}$, followed by two runs in the tetragonal phase at $250\,\mathrm{K}$ and $150\,\mathrm{K}$, and finally one run in the orthorhombic phase at $150\,\mathrm{K}$. All these runs are performed in an *NpT* ensemble with Parinello-Rahman dynamics with friction coefficients set to 3 ps^{-1} for all atomic species and lattice degrees of freedom.

A total of 1132 structures are sampled, and DFT single-point calculations are performed on all these structures using the same DFT parameters as in SI note 1 to generate the final training and validation sets.

An Allegro model is trained using the same model parameters as in SI note 1, with the training and validation sets consisting of 905 and 227 structures, respectively, and the training ran for 1134 epochs. The RMSE in energy and forces during model training and model validation are given in Figure S23. The final model has the training errors of $0.25\,\mathrm{meV/atom}$ in energy and $5.04\,\mathrm{meV/Å}$ in forces, and validation errors of $0.25\,\mathrm{meV/atom}$ in energy and $11.7\,\mathrm{meV/Å}$ in forces.

The accuracy and transferability of this model are validated by performing heating runs from $100\,\mathrm{K}$ to $400\,\mathrm{K}$ for $0.6\,\mathrm{ns}$ using a time step of $1\,\mathrm{fs}$ and mass of H of $1\,\mathrm{amu}$ on $3{\times}3{\times}2$ orthorhombic supercells (864 atoms) of $MAPbI_3$. The window-averaged pseudo-cubic lattice vectors with the window lengths $5\,\mathrm{ps}$ from this run are given in Figure S24. The overall phase diagram closely resembles that observed in experiments.[22]

This NNP is used to perform constant temperature MD runs in an *NpT* ensemble using $6 \times 6 \times 6$ cubic supercells (2592 atoms) of $MAPbI_3$ at different temperatures between $300\,\mathrm{K}$ and $450\,\mathrm{K}$ with steps of $50\,\mathrm{K}$ to obtain equilibrated lattice constants. These values are fitted

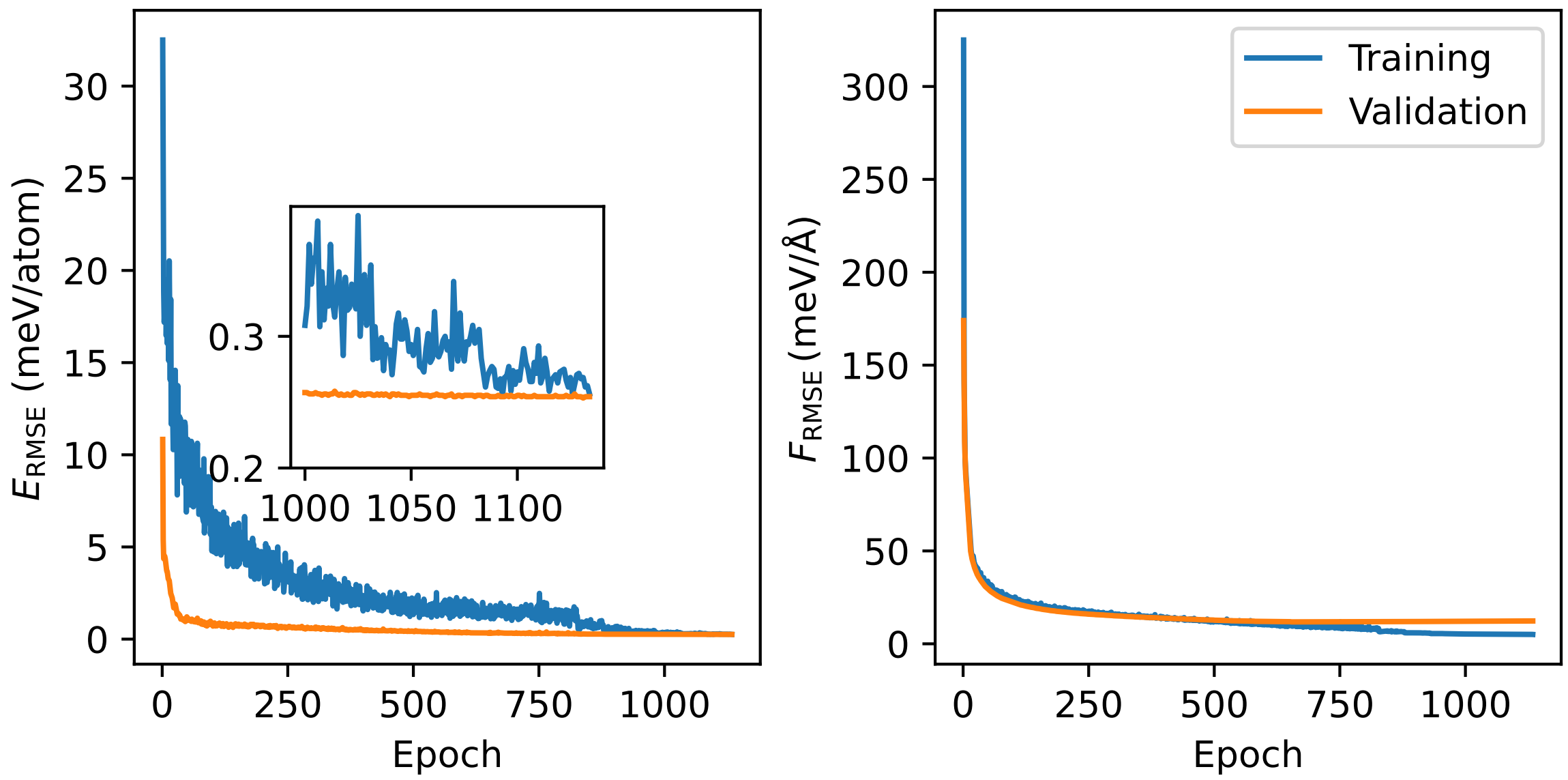

Figure S23: Root mean squared errors in energy (E_{RMSE}) and forces (F_{RMSE}) during model training and model validation for the $MAPbI_3$ phase transition model.

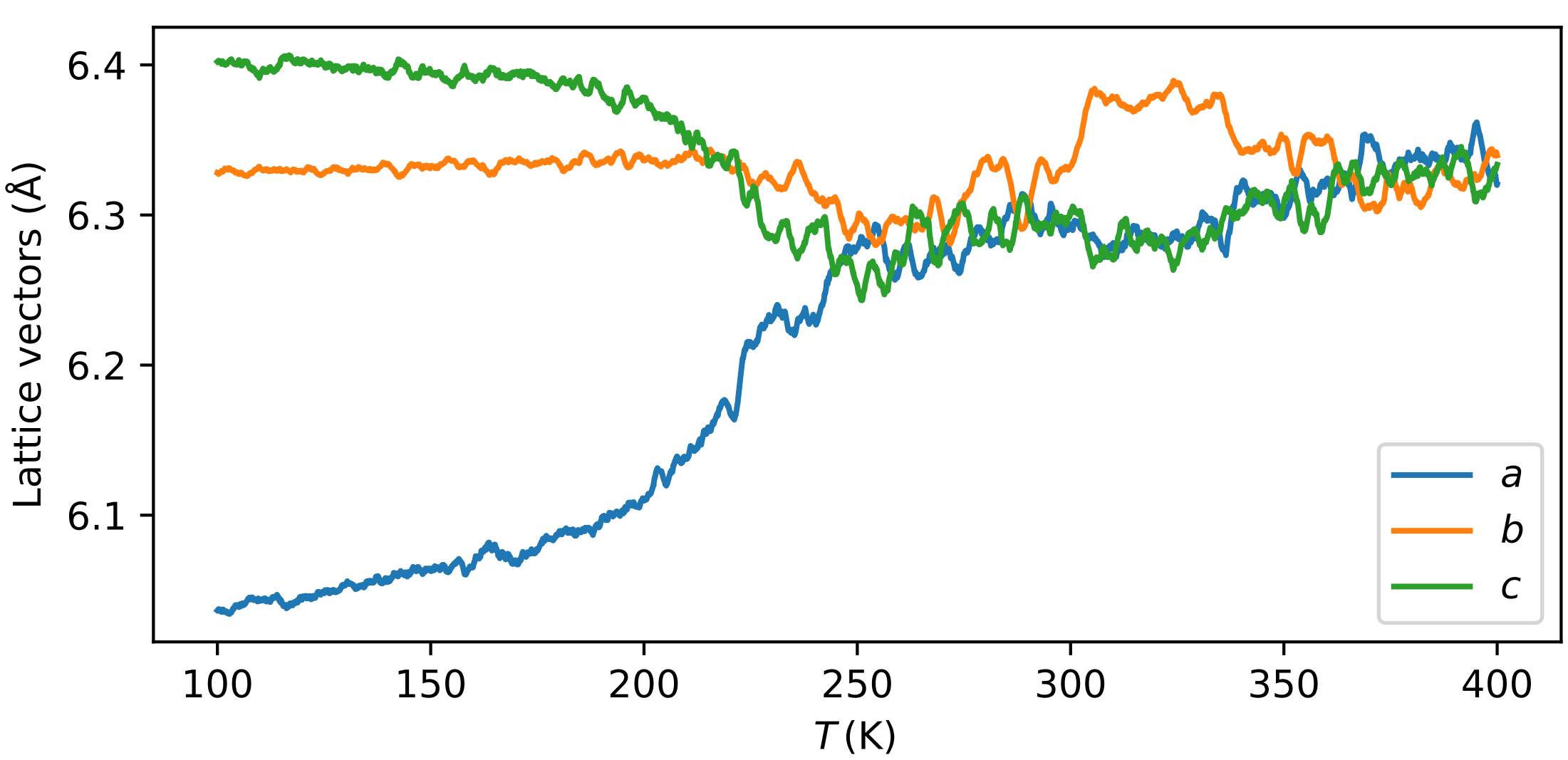

Figure S24: Pseudo-cubic unit cell lattice vectors of $MAPbI_3$ as functions of temperature in the heating MD runs performed using the phase transition NNP.

using linear least-squares regression (Figure S25) and extrapolated to obtain equilibrated volumes between $500\,\mathrm{K}$ and $600\,\mathrm{K}$.

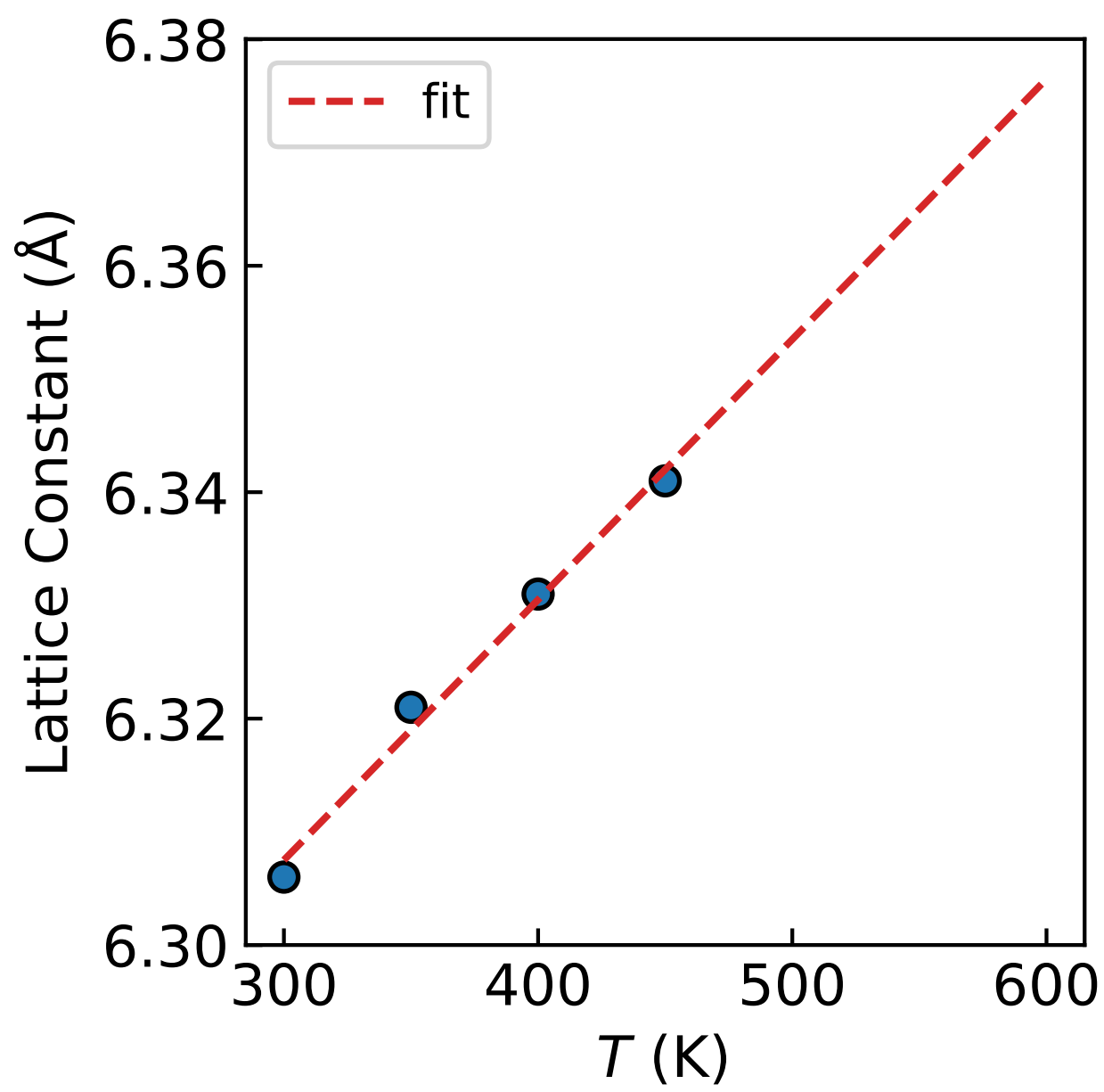

Figure S25: The lattice constant of cubic $MAPbI_3$ as a function of temperature extracted using the phase transition NNP. The blue points represent calculated values, and the dotted red line represents the fit.

4 Production runs and diffusion coefficients

$2\,\mathrm{ns}$ long MD runs are performed at different temperatures between $500\,\mathrm{K}$ and $600\,\mathrm{K}$ using $6 \times 6 \times 6$ supercells $MAPbI_3$ (2592 atoms) with one MA or I point defect in LAMMPS.[23] The lattice constants are obtained using the procedure described in SI note 3, and are given in Table S3.

Table S3: Lattice constants of $MAPbI_3$ unit cell at different temperatures in cubic phase.

Temperature (K)	Lattice constants (Å)
500	6.354
525	6.359
550	6.365
575	6.371
600	6.377

The structures are first equilibrated to the target temperature for $100\,\mathrm{ps}$ with timesteps of $1\,\mathrm{fs}$ in an *NVT* ensemble using a Langevin thermostat with the relaxation time set to $0.33\,\mathrm{ps}$. Following equilibration, $2\,\mathrm{ns}$ long production runs with timesteps of $1\,\mathrm{fs}$ are performed in an *NVT* ensemble using a Nose-Hoover thermostat with the relaxation time set to $0.1\,\mathrm{ps}$ and mass of H to $1\,\mathrm{amu}$. To ensure proper sampling of diffusion coefficients, 3 runs are performed at each temperature. To quantify the migration behavior of the defects, the mean squared displacement (MSD) is plotted over time for each atomic species using the MDAnalysis Python library.[24,25] The MSD plots for $I_{MA}^{+}$and I_{I}^{-} are given in Figure S26 and Figure S27, respectively. The MSD is calculated using

$$\mathrm{MSD}(r_d) = \left\langle \frac{1}{N} \sum_{i=1}^{N} |r_d - r_d(t_0)|^2 \right\rangle_{t_0}, \tag{S1}$$

where N is the number of atoms of a particular atomic species, and r_d are their coordinates in d dimensions (3 for our systems). The Diffusion coefficient D is calculated using

$$D = \frac{N}{2d} \lim_{t \to \infty} \frac{d}{dt} \mathrm{MSD}(r_d), \tag{S2}$$

which is proportional to the slope of the MSD curve, where the factor N ensures that the diffusion coefficient is defect concentration independent. The diffusion coefficient of the MA molecule is

calculated as the average of the diffusion coefficients of C,N, and H (Figure S26).

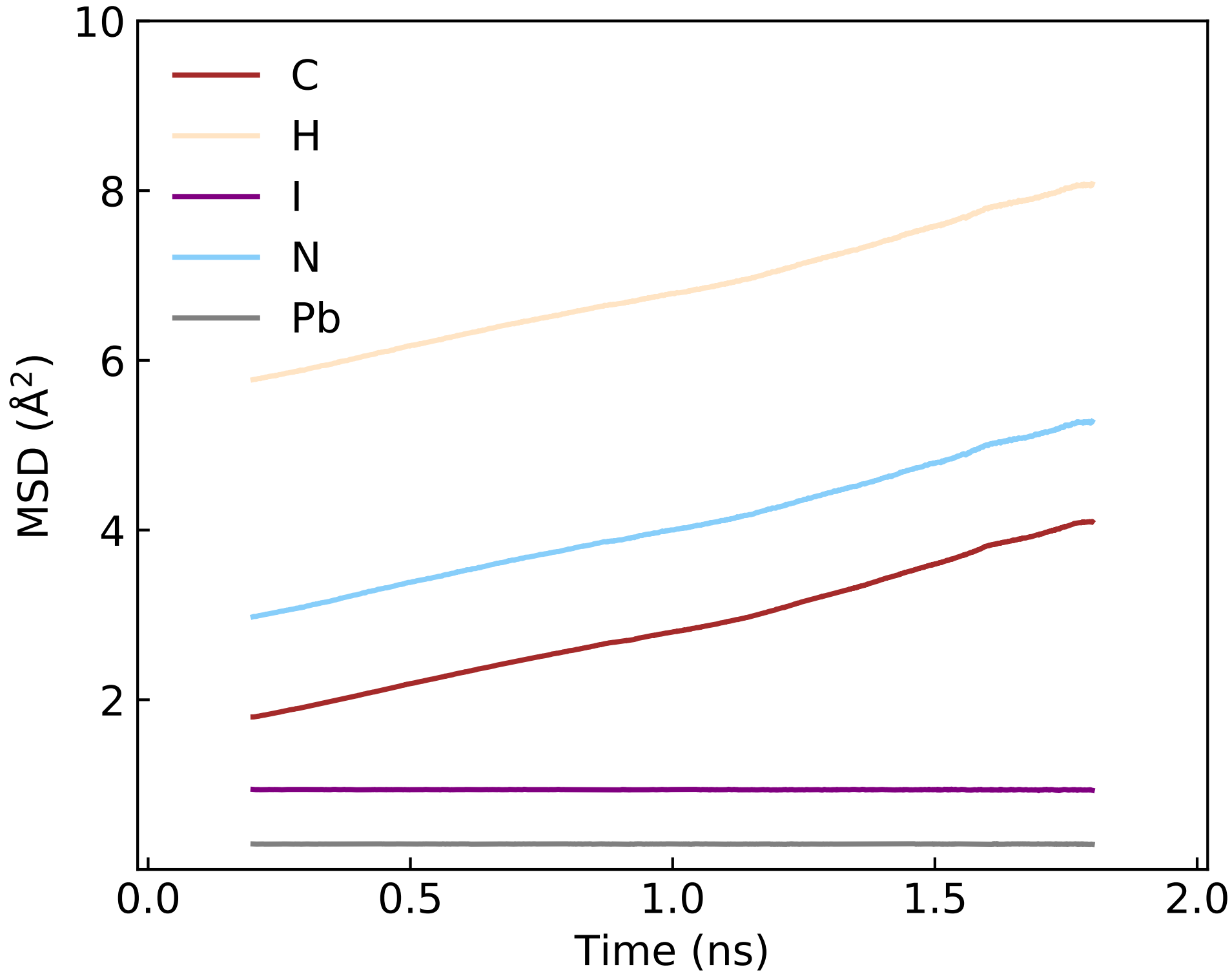

Figure S26: Mean squared displacement (MSD) curves decomposed to the atomic species over simulation time for I_{MA}^{+}in $MAPbI_3$ at $550\,K$.

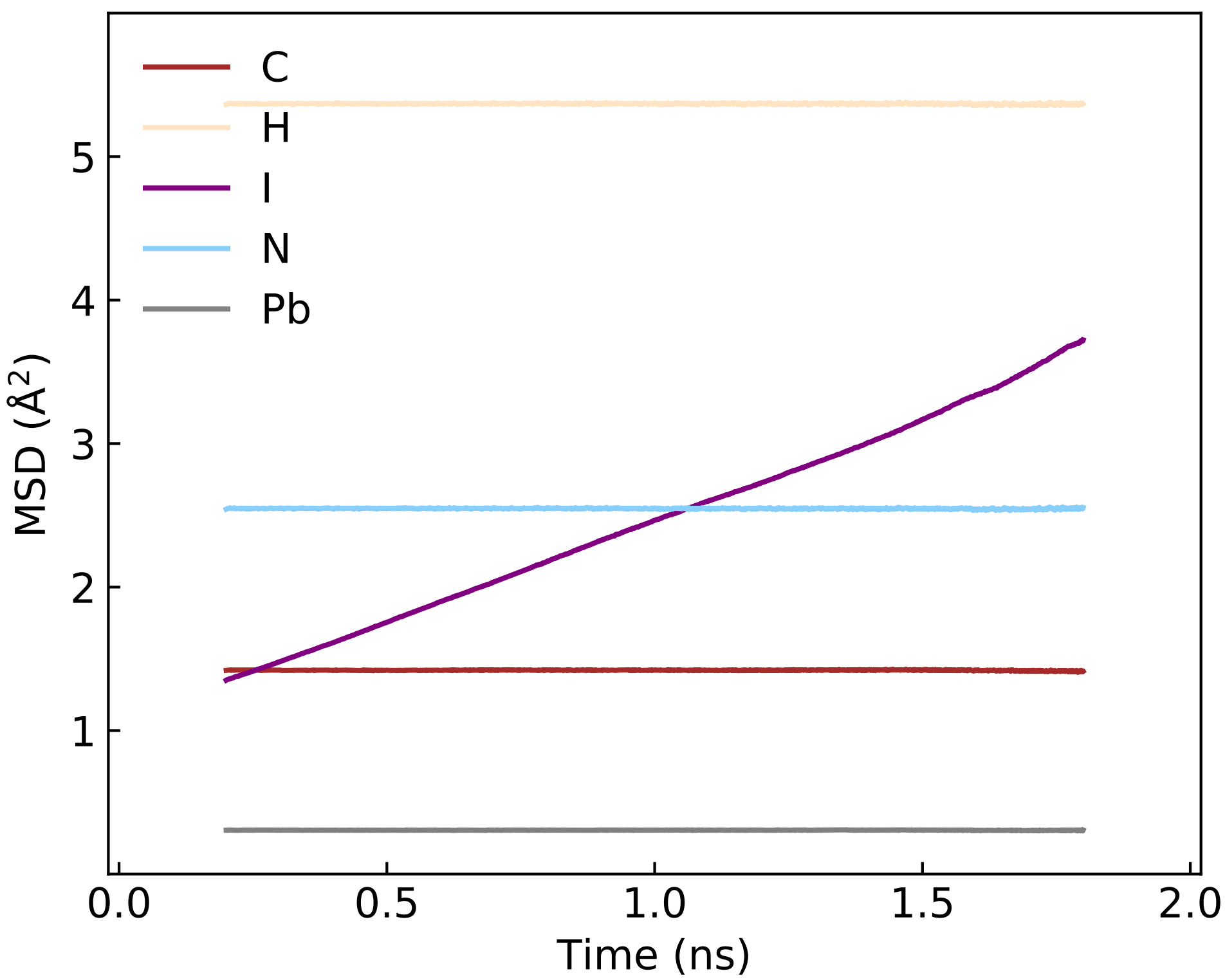

Figure S27: Mean squared displacement (MSD) curves decomposed to the atomic species over simulation time for I_I^- in $MAPbI_3$ at $550\,K$.

5 Charge density analysis

To check the charge distribution of the extra electron in I_{MA}^0, its charge density is compared with that of its positive counterpart. The charge densities of the neutral and positive charge states are calculated using the optimized geometry of I_{MA}^0. The difference between the charge densities is visualized in VESTA,[18] and is given in Figure S28. As evident from the figure, the extra electron delocalizes over the whole lattice.

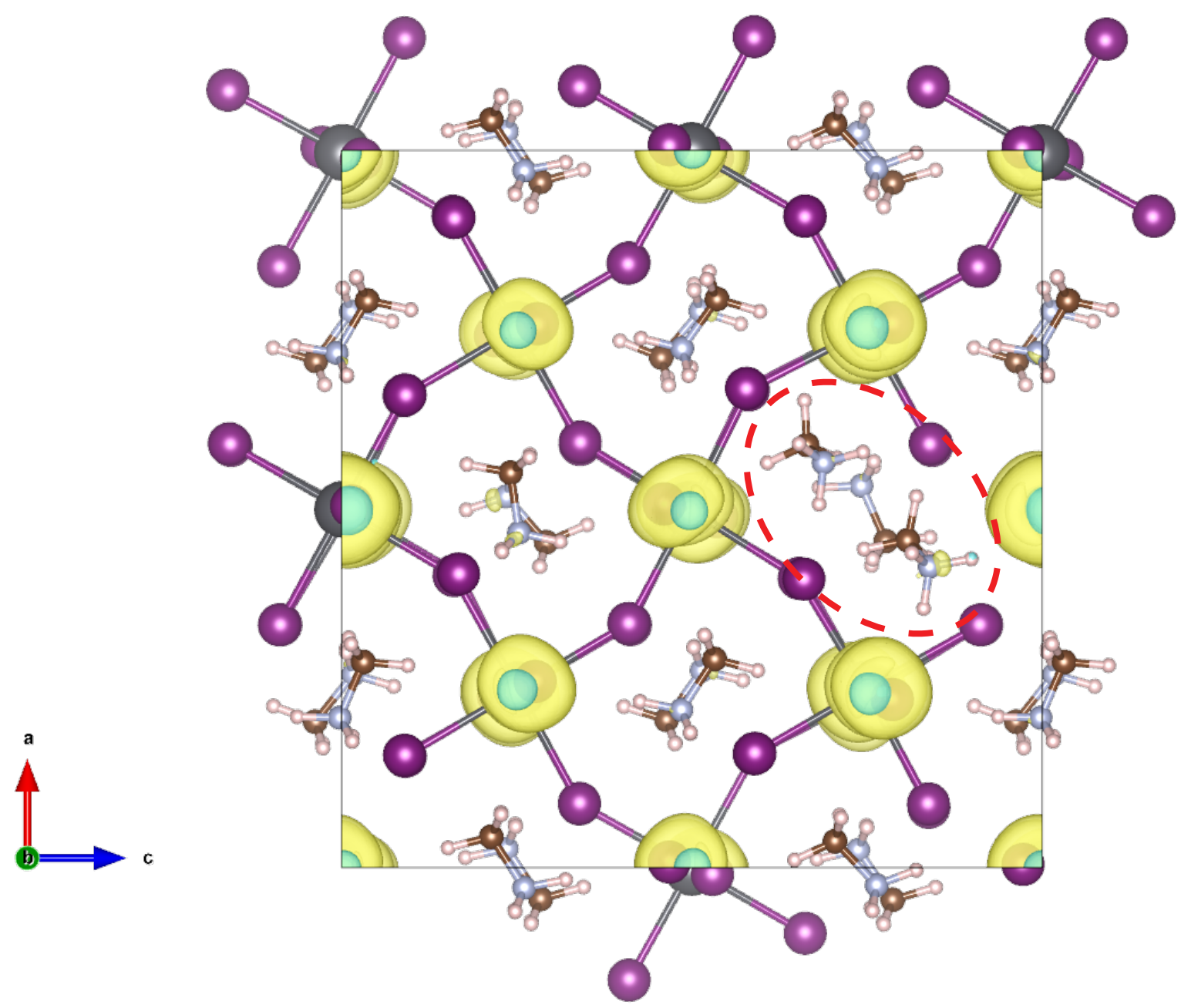

Figure S28: The charge density of the extra electron in I_{MA}^0. The regions with a negative charge are colored in yellow, and the regions with a positive charge are colored in blue. The defect environment is circled in dotted red lines.

6 MA orientation during I_{MA} migration

The relative orientation of the MA molecules during I_{MA} migration events is computed. Two I_{MA} migration events are extracted from a $600\,\mathrm{K}$ MD run, and the angle between the C−N axis of the migrating MA molecule and that of the MA molecule in the Pb−I cage it migrates from, and that of the MA molecule in the Pb−I cage it migrates to (Figure S29), are computed. These angles along the migration events are given in Figure S30. As is evident from the figure, the I_{MA} migration events occur on ps timescales. At the start of the migration event, the migrating MA molecule is aligned roughly parallel to the neighbouring MA molecule in the cage it is migrating to. As the migrating molecule enters that cage, it pushes the molecule that is already there to a perpendicular orientation before the two end up in a roughly parallel orientation in that cage. The orientation of the molecule that remains in the cage where the migrating molecule is jumping from is less connected to the latter molecule. It fluctuates, but follows the motion somewhat.

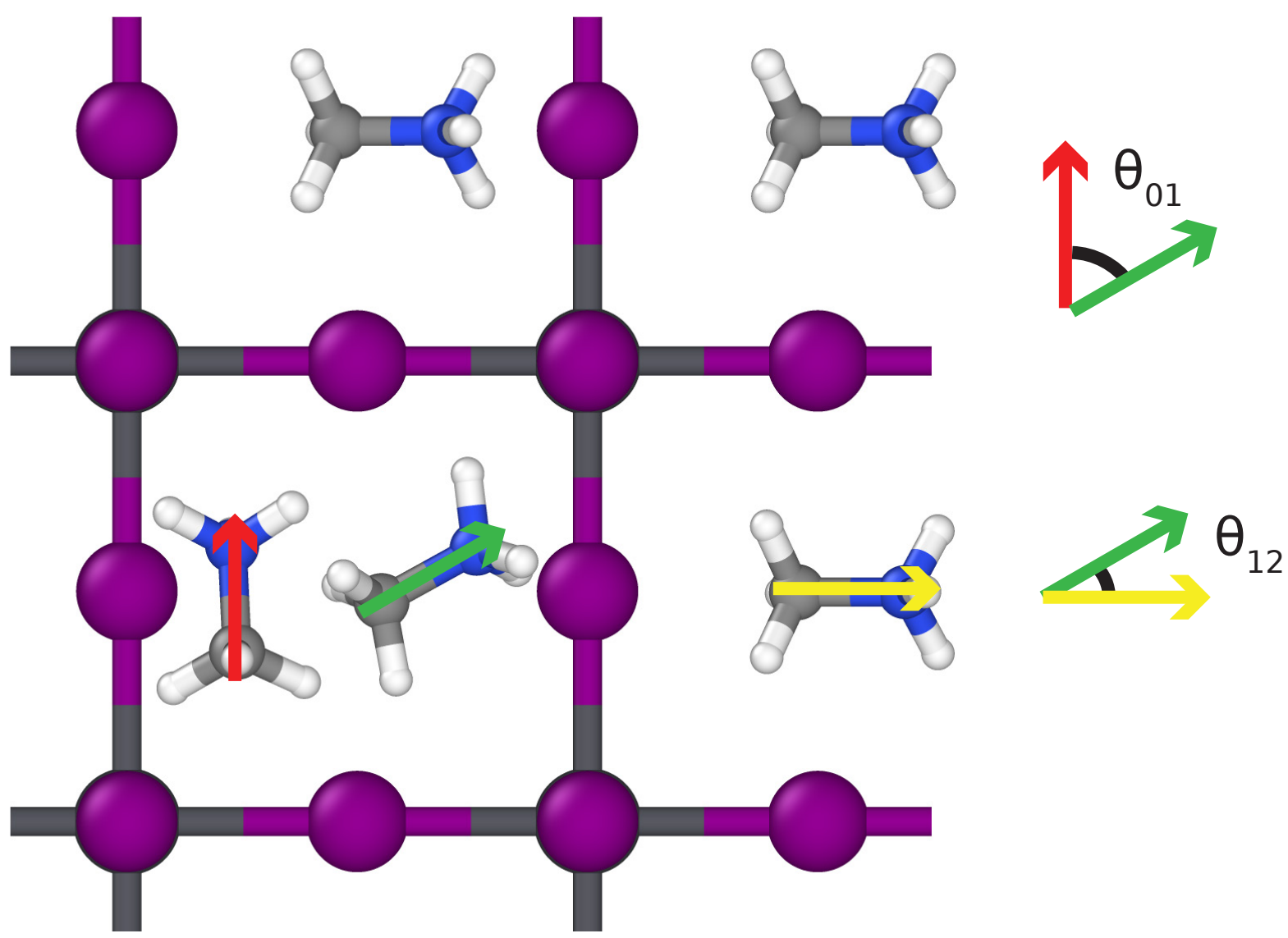

Figure S29: Schematic representation of the relative angles between the three MA molecules during I_{MA} migration events. The green arrow represents the C−N axis of the migrating MA molecule, the red arrow represents the C−N axis of the MA molecule in the Pb−I cage it migrates from and the yellow arrow represents the C−N axis of the MA molecule in the Pb−I cage it migrates to.

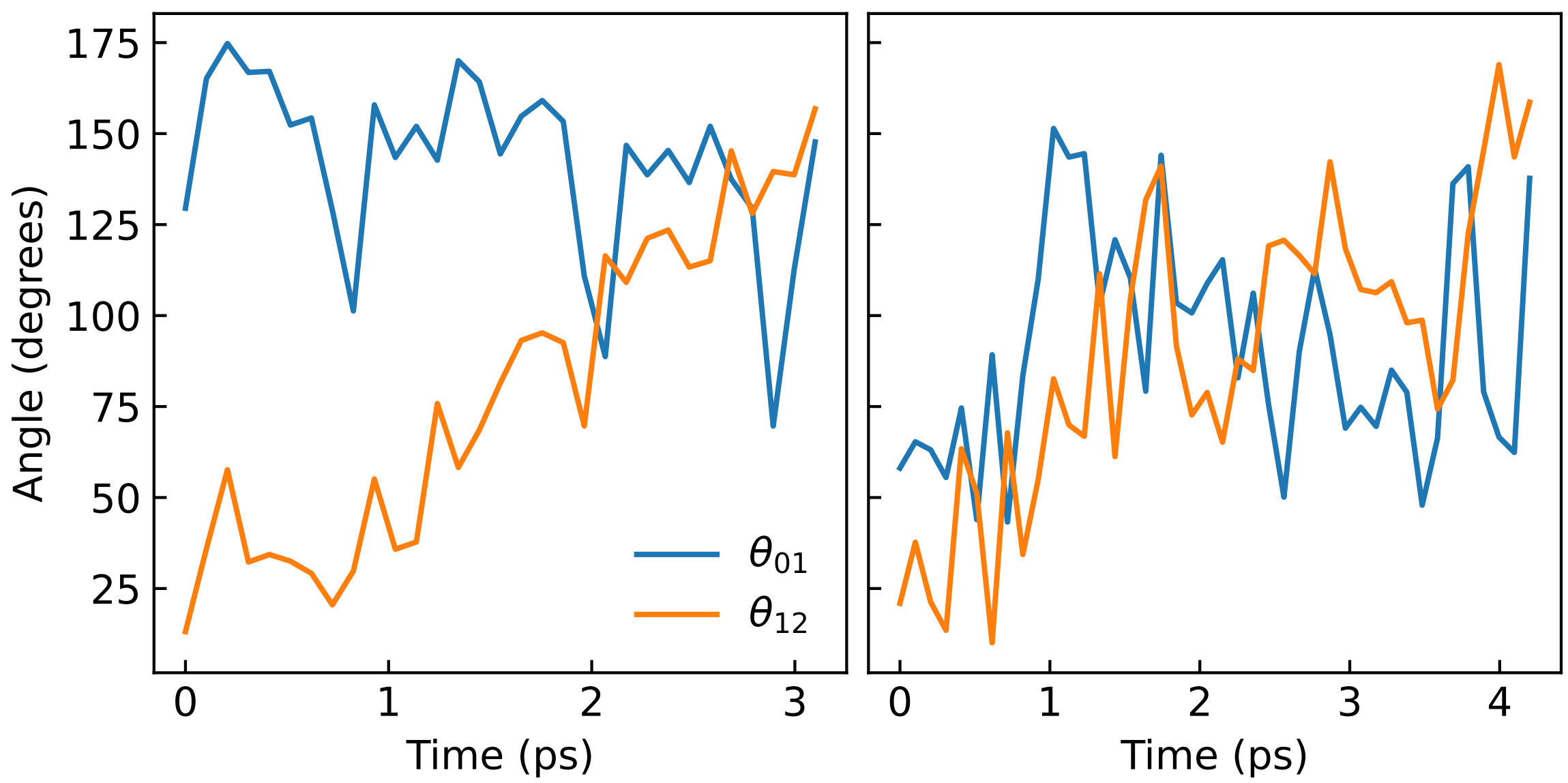

Figure S30: The angle between the C−N axis of the migrating MA molecule and that of the MA molecule in the Pb−I cage it migrates from (θ_{01}), and with that of the MA molecule in the Pb−I cage it migrates to (θ_{12}) along two I_{MA} migration events extracted from a $600\,\mathrm{K}$ MD run.

References

(1) Kresse, G.; Furthmüller, J. Efficient iterative schemes for ab initio total-energy calculations using a plane-wave basis set. *Phys. Rev. B* **1996**, *54*, 11169–11186.

(2) Jinnouchi, R.; Karsai, F.; Kresse, G. On-the-fly machine learning force field generation: Application to melting points. *Phys. Rev. B* **2019**, *100*, 014105.

(3) Jinnouchi, R.; Lahnsteiner, J.; Karsai, F.; Kresse, G.; Bokdam, M. Phase Transitions of Hybrid Perovskites Simulated by Machine-Learning Force Fields Trained on the Fly with Bayesian Inference. *Phys. Rev. Lett.* **2019**, *122*, 225701.

(4) Bartók, A. P.; Payne, M. C.; Kondor, R.; Csányi, G. Gaussian Approximation Potentials: The Accuracy of Quantum Mechanics, without the Electrons. *Phys. Rev. Lett.* **2010**, *104*, 136403.

(5) Bartók, A. P.; Kondor, R.; Csányi, G. On representing chemical environments. *Phys. Rev. B* **2013**, *87*, 184115.

(6) Parrinello, M.; Rahman, A. Crystal Structure and Pair Potentials: A Molecular-Dynamics Study. *Phys. Rev. Lett.* **1980**, *45*, 1196–1199.

(7) Parrinello, M.; Rahman, A. Polymorphic transitions in single crystals: A new molecular dynamics method. *J. Appl. Phys.* **1981**, *52*, 7182–7190.

(8) Kresse, G.; Joubert, D. From ultrasoft pseudopotentials to the projector augmented-wave method. *Phys. Rev. B* **1999**, *59*, 1758–1775.

(9) Sun, J.; Ruzsinszky, A.; Perdew, J. P. Strongly Constrained and Appropriately Normed Semilocal Density Functional. *Phys. Rev. Lett.* **2015**, *115*, 036402.

(10) Sabatini, R.; Gorni, T.; de Gironcoli, S. Nonlocal van der Waals density functional made simple and efficient. *Phys. Rev. B* **2013**, *87*, 041108.

(11) Lahnsteiner, J.; Kresse, G.; Heinen, J.; Bokdam, M. Finite-temperature structure of the $MAPbI_3$ perovskite: Comparing density functional approximations and force fields to experiment. *Phys. Rev. Mater.* **2018**, *2*, 073604.

(12) Bokdam, M.; Lahnsteiner, J.; Ramberger, B.; Schäfer, T.; Kresse, G. Assessing Density Functionals Using Many Body Theory for Hybrid Perovskites. *Phys. Rev. Lett.* **2017**, *119*, 145501.

(13) Xue, H.; Brocks, G.; Tao, S. First-principles calculations of defects in metal halide perovskites: A performance comparison of density functionals. *Phys. Rev. Mater.* **2021**, *5*, 125408.

(14) Monkhorst, H. J.; Pack, J. D. Special points for Brillouin-zone integrations. *Phys. Rev. B* **1976**, *13*, 5188–5192.

(15) Batzner, S.; Musaelian, A.; Sun, L.; Geiger, M.; Mailoa, J. P.; Kornbluth, M.; Molinari, N.; Smidt, T. E.; Kozinsky, B. E(3)-equivariant graph neural networks for data-efficient and accurate interatomic potentials. *Nat. Comm.* **2022**, *13*, 2453.

(16) Musaelian, A.; Batzner, S.; Johansson, A.; Sun, L.; Owen, C. J.; Kornbluth, M.; Kozinsky, B. Learning local equivariant representations for large-scale atomistic dynamics. *Nat. Comm.* **2023**, *14*, 579.

(17) Henkelman, G.; Uberuaga, B. P.; Jónsson, H. A climbing image nudged elastic band method for finding saddle points and minimum energy paths. *The Journal of Chemical Physics* **2000**, *113*, 9901–9904.

(18) Momma, K.; Izumi, F. *VESTA*: a three-dimensional visualization system for electronic and structural analysis. *Journal of Applied Crystallography* **2008**, *41*, 653–658.

(19) Lahnsteiner, J.; Bokdam, M. Anharmonic lattice dynamics in large thermodynamic ensem-

bles with machine-learning force fields: $CsPbBr_3$, a phonon liquid with Cs rattlers. *Phys. Rev. B* **2022**, *105*, 024302.

(20) Tyagi, V.; Pols, M.; Brocks, G.; Tao, S. Tracing Ion Migration in Halide Perovskites with Machine Learned Force Fields. *J. Phys. Chem. Lett.* **2025**, *16*, 5153–5159.

(21) Peng, A.; Cai, C.; Guo, M.; Zhang, D.; Zhang, C.; Jiang, W.; Wang, Y.; Loew, A.; Wu, C.; E, W. et al. LAMBench: a benchmark for large atomistic models. *Npj Comput. Mater.* **2026**, *12*, 62.

(22) Whitfield, P. S.; Herron, N.; Guise, W. E.; Page, K.; Cheng, Y. Q.; Milas, I.; Crawford, M. K. Structures, Phase Transitions and Tricritical Behavior of the Hybrid Perovskite Methyl Ammonium Lead Iodide. *Scientific Reports* **2016**, *6*, 35685.

(23) Thompson, A. P.; Aktulga, H. M.; Berger, R.; Bolintineanu, D. S.; Brown, W. M.; Crozier, P. S.; in 't Veld, P. J.; Kohlmeyer, A.; Moore, S. G.; Nguyen, T. D. et al. LAMMPS - a flexible simulation tool for particle-based materials modeling at the atomic, meso, and continuum scales. *Comp. Phys. Comm.* **2022**, *271*, 108171.

(24) Michaud-Agrawal, N.; Denning, E. J.; Woolf, T. B.; Beckstein, O. MDAnalysis: A toolkit for the analysis of molecular dynamics simulations. *J. Comput. Chem.* **2011**, *32*, 2319–2327.

(25) Maginn, E. J.; Messerly, R. A.; Carlson, D. J.; Roe, D. R.; Elliot, J. R. Best Practices for Computing Transport Properties 1. Self-Diffusivity and Viscosity from Equilibrium Molecular Dynamics [Article v1.0]. *LiveCoMs* **2018**, *1*, 6324.
